# Seed Diversity in the Tribe Miconieae (Melastomataceae): Taxonomic, Systematic, and Evolutionary Implications

**DOI:** 10.1371/journal.pone.0100561

**Published:** 2014-06-23

**Authors:** Gilberto Ocampo, Fabián A. Michelangeli, Frank Almeda

**Affiliations:** 1 California Academy of Sciences, Institute for Biodiversity Science and Sustainability, Department of Botany, San Francisco, California, United States of America; 2 The New York Botanical Garden, Bronx, New York, United States of America; The National Orchid Conservation Center of China; The Orchid Conservation & Research Center of Shenzhen, China

## Abstract

Miconieae is the largest tribe in the Melastomataceae with over 1,850 species. The members of Miconieae display a wide range of morphological diversity, and seed morphology is no exception. Previous studies have found that seed morphological diversity is not congruent with traditional classifications, and suggest that it may reflect evolutionary relationships within Miconieae. Here we characterize seed morphology of 364 species of Miconieae. The morphological data set and a DNA sequence data matrix were analyzed under a parsimony and Bayesian framework. Seed characters were used to test taxonomic and clade hypotheses, to estimate morphological ancestral character states, and to assess phylogenetic signal. The phylogenetic analyses of morphological data retrieved a poorly-resolved, low-supported phylogeny; in contrast, a relatively strongly supported phylogeny was estimated using the molecular data. Hypothesis testing procedures could only reject the monophyly of *Clidemia*, *Leandra*, and *Miconia*. The results indicated that the seed morphological characters were homoplasious, but contained phylogenetic signal. The morphological seed types that were described in previous studies did not support any of the clades retrieved by the molecular phylogeny. In contrast with previous investigations, our study shows that although seed morphology is very variable, it does not provide information for supporting some genera or clades within Miconieae. However, it is suggested that seed characters in combination with other vegetative and reproductive traits may aid in the characterization of smaller clades. The presence of phylogenetic signal retrieved by homoplasious characters may indicate that diversification of seed characters could have an adaptive component. Further studies that increase taxon sampling, refine seed trait characterization, and evaluate the alleged relationships between environmental variables and seed diversification will contribute to a better understanding of seed morphology and evolution in this species-rich tribe.

## Introduction

Seed morphology has long been considered an important source of character information for taxonomic purposes and has been proposed as a feature that reflects the evolutionary history of plants [1]. Seed size, shape, and epidermal surface features have figured prominently in the characterization of seed morphological diversity [2], [3], and it has been argued that these seed characters may provide data for circumscribing taxa at different taxonomic levels [1], [2]. While some studies support this hypothesis [4], [5], other investigations show that the systematic and taxonomic value of seed micromorphology may be limited [6]–[9].

The Melastomataceae are one of the largest flowering plant families [10], with 166–179 genera and over 5,400 species mainly distributed in tropical and subtropical areas of the world [11]–[13]. The vegetative and reproductive characters are very diverse across the family [10], [14], and seed morphology is no exception. Seed morphological features have been used in the past for circumscribing a number of infrafamilial taxa [15]–[18]. However, more recent investigations have found that seed morphological traits do not always correspond to proposed classifications [19]–[22]. These studies suggested that the delimitation of some tribes and genera should be reconsidered using seed morphology based on the assumption that seed morphology yields information about the evolutionary history of the groups under study.

The Miconieae, one of some 20 tribes in the Melastomataceae (Darin Penneys et al., unpublished data), comprises over 1,850 species and ca. 17 genera restricted to the New World [23]. Analyses of DNA sequence data have shown that all but one of the genera of Miconieae, as currently circumscribed, are not monophyletic [23]–[26], supporting the notion that they are ambiguously defined and are frequently difficult to distinguish [18], [27]–[30]. Seed morphological features in the tribe are diverse and variable [31], but they do not support established classifications at generic and sectional levels [22], [26]. However, some studies that incorporated a phylogenetic framework have identified a number of seed characters that are potential synapomorphies for certain clades [26], [32], but taxon sampling remains insufficient for drawing definitive conclusions for the Miconieae as a whole. In addition, described morphological seed types that are often not interchangeable among studies make comparative analyses a difficult task (compare [22], [26], [33]).

The aim of this investigation is to increase taxon sampling in the Miconieae to evaluate the taxonomic and systematic value of seed morphological characters in a phylogenetic context. Due to the existence of different morphological types proposed for Miconieae and the potential problems of composite coding [34], we have used the terminology proposed by Ocampo and Almeda [31]. This allowed us to test the monophyly of the genera traditionally recognized within the tribe [18], [27], [28], and the clades recovered in recent molecular phylogenetic studies [23], [25]. It has also provided a standard to help hypothesize about the evolutionary transitions of seed traits.

## Materials and Methods

### Taxon sampling and data acquisition

The sampled taxa represent all major clades of Miconieae recovered in the studies of Michelangeli et al. [23] and Goldenberg et al. [25]. A total of 390 species, including 26 outgroup taxa, were considered in this investigation (Table S1). Species of the tribe Merianieae and a clade composed of *Eriocnema* + *Physeterostemon* have been recovered as sister groups of Miconieae [25], [35]; therefore, taxa representing those lineages were selected to serve as outgroups. Seed sample preparation, scanning electron microscopy (SEM) image acquisition, and seed length measurements were done at the California Academy of Sciences (CAS) and the New York Botanical Garden (NY), following the procedures described by Ocampo and Almeda [31]. Seed morphological information for additional taxa was taken mainly from Ocampo and Almeda [31].

### Character coding

A subset of the characters proposed by Ocampo and Almeda [31] were used to describe seed diversity in Miconieae. Polymorphic characters were allowed. The characters scored for this study were: A) Three-dimensional shape: 0, ovoid, 1, pyramidal; 2, subspheroid; 3, subacerose. B) Base of the body of the seed horizontally expanded: 0, absent; 1, present. C) Location of the highest point perpendicular to the raphal zone: 0, toward the chalazal side; 1, toward the central part of the seed. D) Symmetrical plane of the raphal zone: 0, ovate; 1, triangular; 2, circular; 3, elliptic; 4, obtriangular; 5, suboblong; 6, obovate; 7, linear. E) Length of the raphal zone proportional to the total length of the seed: 0, <70%; 1, 70–85%; 2, 90–100%; 3, >100%. F) Ventrally-oriented expansion of the raphal zone: 0, absent; 1, present. G) Appendage: 0, absent; 1, present. H) Multicellular sculpture: 0, absent; 1, present. I) General arrangement of the cells with respect to each other: 0, irregular; 1, aligned. J) Cell shape: 0, isodiametric; 1, elongate. K) Relief of the anticlinal walls: 0, inconspicuous; 1, channeled; 2, raised. L) Curvature of the anticlinal walls: 0, undulate; 1, irregularly curved. M) Relief of the periclinal walls: 0, flat to convex; 1, par-convex; 2, concave. N) Periclinal walls dividing into two or more segments: 0, absent; 1, present. O) Microrelief of the periclinal walls: 0, absent; 1, present. P) Cells with features differing from the rest of the seed corpus: 0, absent; 1, present. Q) Length of the seed from antiraphal view (mm): 0, <0.5; 1, 0.5–0.99; 2, 1.0–1.49; 3, 1.5–1.99; 4, 2.0–2.49; 5, 2.5–2.99; 6, 3.0–3.49; 7, ≥3.5. Seed images newly generated for this study and a subset of samples included in Ocampo and Almeda [31] were coded with the program Mesquite version 2.75 [36]. The images are available at http://sweetgum.nybg.org/melastomataceae/images.php.

### Phylogenetic analysis

The morphological data matrix was analyzed under Maximum Parsimony (MP) and Bayesian Markov chain Monte Carlo (MCMC) inference [37] in order to detect if the morphological data retrieved the clades recovered in recent molecular phylogenetic studies [23], [25]. MP analyses were run in PAUP* version 4.0 [38] considering all characters as unordered and multistate characters as polymorphic; starting trees were obtained via stepwise addition, and the analyses used a heuristic search strategy with 1,000 random addition sequences, tree-bisection-reconnection (TBR) branch swapping, with the number of rearrangements limited to 10,000,000, max trees  = 10,000, and the results were summarized as a strict consensus tree. Clade support was determined by nonparametric bootstrapping (BS; [39]) from 10,000 replicates with simple addition and TBR branch swapping, holding only one tree per replicate as recommended by Müller [40]. The Bayesian analyses used the standard model for morphological characters as implemented in the program MrBayes version 3.2.1 [41] under the Mkv model [42] and the coding option set to “variable”; two independent analyses were run with 40,000,000 generations each using the MCMC algorithm, trees were sampled every 1,000 generations, and the first 50% of the tree samples were discarded as burn-in for obtaining a 50% majority-rule consensus tree. Clade support was obtained by Bayesian posterior probabilities (p.p.) [43], [44]. All analyses were performed using the computer cluster of the Center for Comparative Genomics at the California Academy of Sciences.

In order to obtain a molecular phylogenetic framework, DNA sequence data for all species under study were retrieved from GenBank. Sequences were aligned using MUSCLE version 3.7 [45], followed by manual alignment, and concatenated for obtaining a combined data set of nuclear (ITS and ETS) and chloroplast (*accD-psaI* and *psbK-psbI* intergenic spacers) DNA loci. Phylogenetic analyses were performed as above, but the Bayesian analysis was conducted using the best-fit model of evolution provided by MrModel test version 2.3 [46] under the Akaike Information Criterion (AIC) [47] for each partition. The model selected for ITS, ETS, and *accD-psaI* was a general time reversible model (GTR) [48] plus parameters for proportion of invariant sites (I) [49] and a gamma-distributed rate variation (G) [50]. For *psbK-psbI* the model selected was GTR + G. Bayesian analysis was run with two replicates for 20,000,000 generations, and the first 50% of sampled trees were discarded for obtaining a 50% majority-rule consensus tree.

Incongruence between the morphological and DNA sequence data was tested using the incongruence length difference (ILD) test [51] as implemented in PAUP* as the partition homogeneity test. The analysis was run as in the MP analyses and with 250 replicates and 25 random addition sequences.

### Hypothesis testing

The seed morphological dataset was used to test the monophyly of the traditional genera in Miconieae [18], [27], [28] and major clades recovered in molecular phylogenetic analyses [23], [25]. Individual constraint trees compatible with genera and clades were created in Mesquite. These trees were loaded into PAUP*, and the analyses were run under the same search strategy as in the MP analysis in order to find the shortest trees consistent with the constraint. Afterwards, a random MP tree and a random MP tree consistent with the constraint were compared using Templeton's test [52].

### Ancestral character reconstruction and phylogenetic signal

Ancestral character reconstruction of the morphological characters was performed in Mesquite using the parsimony criterion and considering the character states as unordered. Morphological data was optimized onto one of the most parsimonious trees and the tree with the highest likelihood that resulted from the MP and Bayesian analyses of the DNA sequence data, respectively. In addition, the morphological data set was loaded in the program MacClade version 4.08a [53] to summarize evolutionary transitions on the aforementioned trees and to detect synapomorphies under a parsimony framework. The analysis was done using the “Trace All Changes” calculation and with the “Unambiguous changes only” option in effect.

Detection of phylogenetic signal (defined by Blomberg and Garland [54] as “a tendency for related species to resemble each other more than they resemble species drawn at random”) of the morphological data was performed in Mesquite, using as references both the MP and the Bayesian trees mentioned above. For each reference tree, 10,000 trees were generated by randomization of terminal taxa, and the number of steps was calculated for all of them under the parsimony criterion. The probability that the data does not have phylogenetic signal is obtained by the number of trees with equal or lower number of steps than the reference tree divided by 10,000; if *P*<0.05, then the data were considered to have significant phylogenetic signal [55].

### Distribution of morphological seed types in the phylogeny

The species in our sampling that have been associated with a morphological type were identified in the phylogeny. The morphotypes follow the classifications proposed by Groenendijk et al. [22] and Martin and Michelangeli [33].

## Results

### Phylogenetic analyses

**Figure 1:**
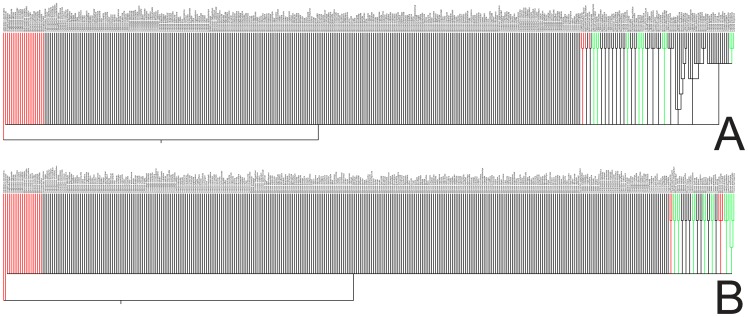
Phylogenetic relationships of species of Miconieae estimated from analyses of seed morphological data. A) Maximum parsimony (MP) strict consensus tree. B) 50% majority-rule Bayesian consensus tree. Nodal support for relationships among the samples was <75% bootstrap and <0.95 posterior probabilities (p.p.) for the MP and Bayesian analyses, respectively. Red branches  =  outgroup taxa; green branches  =  clade present in both analyses.

The morphological data matrix had 3.8% of missing data; nine characters have polymorphic data, which corresponded to 2.5% of the data set (Table S2). Statistics for the MP analysis and consensus trees from the MP and Bayesian runs of the morphological data are shown in Table 1 and Figure 1, respectively. Few clades were recovered in both analyses, and only seven of them were found in both MP and Bayesian trees. However, all the relationships had low support values (BS<75% and p.p.<0.95).

**Table 1:** Statistics for the maximum parsimony analyses of the morphological and DNA sequence data sets.

Statistic	Morphology data	DNA sequence data
Number of most parsimonious trees	1024	3840
Tree length	526	7514
Aligned length	17	3052
Variable sites (proportion)	17 (1)	1615 (0.53)
Parsimony informative sites (proportion)	17 (1)	1058 (0.35)
Ensemble consistency index	0.386	0.377
Ensemble retention index	0.744	0.741
Rescaled consistency index	0.287	0.279

**Figure 2:**
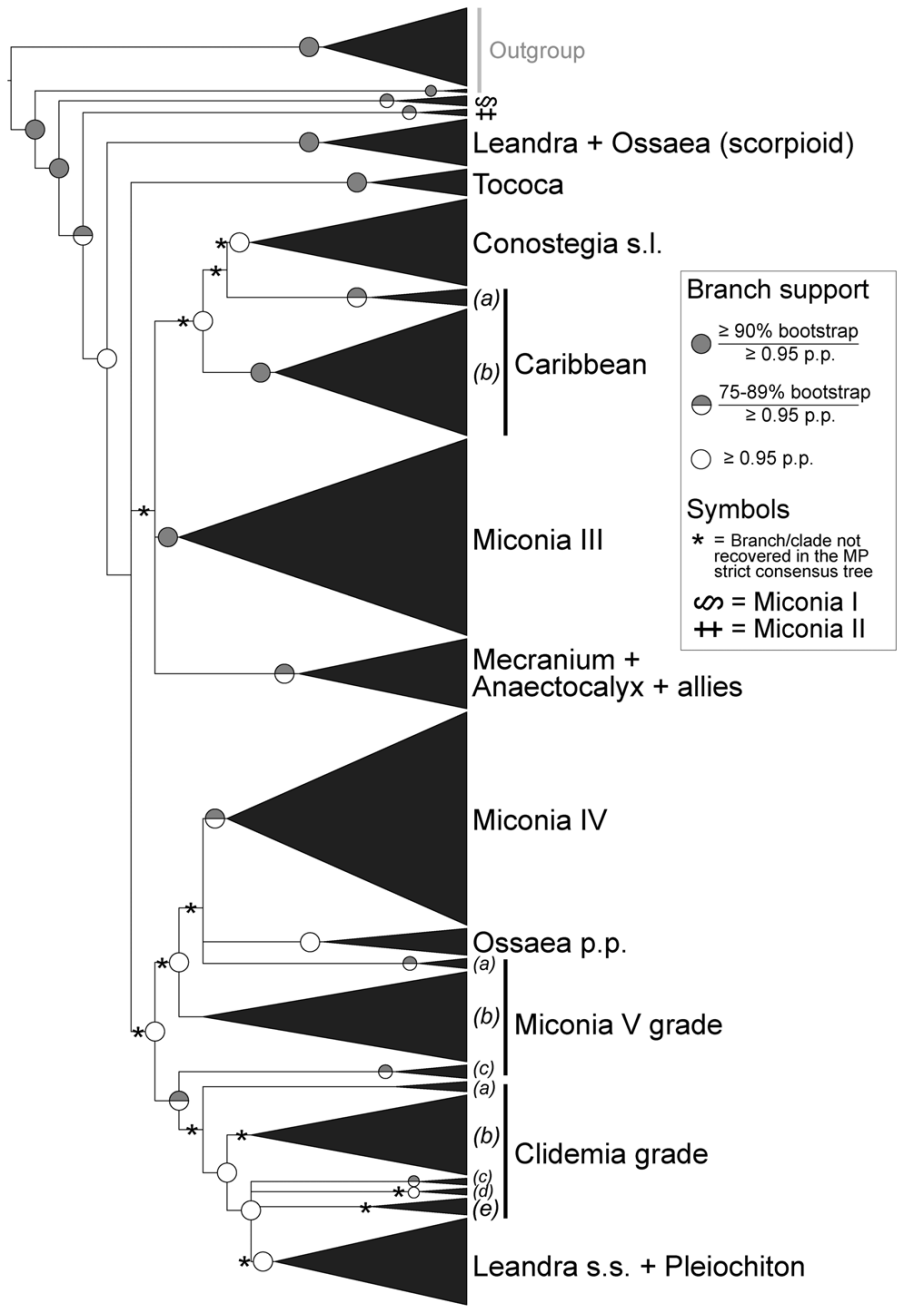
Phylogenetic relationships of major clades in Miconieae estimated from analyses of a combined data matrix of ETS, ITS, *accD-psaI* and *psbK-psbI* DNA sequences. Bayesian 50% majority-rule consensus tree. Nodes with bootstrap support ≥75% and posterior probabilities ≥0.95 are indicated. Clade names were adopted, with some modifications, from Goldenberg et al. [25]; single letters within parentheses are used to label groups within the “Caribbean” clade and the “Clidemia V” and “Miconia V” grades.

A summary of the phylogenetic relationships among major clades inferred from the DNA sequence data is shown in Figure 2 (a Bayesian 50% majority-rule consensus tree with a SEM image of the seed of each species is shown in Figure S1) and the statistics for the MP analysis are summarized in Table 1. Clade names were adopted, with some modifications, from Goldenberg et al. [25]. The MP and Bayesian analyses recovered similar topologies, although the MP strict consensus tree had lower resolution for the relationships among the major clades within Miconieae. All major monophyletic groups had BS support of ≥75% and/or p.p.≥0.95, except for clades (*b*) and (*e*) of the “Clidemia grade”, and the “Miconia V grade (*b*)” clade.

The ILD test showed that the morphological and DNA sequence data sets were significantly incongruent (*P*<0.05). However, because the test has been shown to be problematic [56], a combined data matrix with molecular and morphological data was prepared for exploring variations in nodal support for major clades. The resulting phylogenies were nearly identical to the trees retrieved by the molecular data and clade support did not improve (results not shown).

The topologies of the parsimony and Bayesian consensus trees of morphological and DNA sequence data analyses were highly incongruent. Only the sister relationship between *Clidemia allardii* and *C. crenulata* [“Clidemia grade (*b*)”] was consistently present in all of the trees. The MP analysis of morphological seed data recovered all species of the “Leandra + Ossaea (scorpioid)” clade except *Ossaea capillaris*, but included *C. pustulata* [“Clidemia grade (*b*)”] as a member of this clade. All other relationships derived from the MP run were not consistent with the topology retrieved by the DNA data. In contrast, the Bayesian analysis of the morphological data set recovered a limited number of relationships between pairs of species that are members of the “Clidemia grade (*c*)” clade (*Leandra aristigera* +*L. chaetodon*), “Miconia IV” clade (*Miconia hyemalis* +*M. lymanii*), and the “Ossaea p.p.” clade (*Clidemia radicans* +*C. reitziana*; *Ossaea brenesii* +*O. micrantha*; and *O. macrophylla* +*O. spicata*).

### Hypothesis testing

Results of the Templeton tests are shown in Table 2. The test only rejected the monophyly of the traditionally recognized genera *Leandra* and *Miconia*, and *Clidemia* had a marginal value of *P* = 0.0506. All other topological hypotheses representing genera and major clades could not be rejected.

**Table 2:** Templeton test results using seed morphological data for evaluation of monophyly of traditional genera and clades recognized in Miconieae.

Hypothesis	Difference in tree length between MP trees and those consistent with the constraint	*z* value	*P*	Outcome
**TAXONOMIC CONSTRAINTS**				
*Calycogonium* monophyletic	+10	−0.441	0.659	Cannot reject
*Charianthus* monophyletic	+3	−0.026	0.972	Cannot reject
*Clidemia* monophyletic	+31	−1.954	0.0506	Cannot reject
*Conostegia* monophyletic	+9	−0.342	0.732	Cannot reject
*Leandra* monophyletic	+25	−2.154	0.031	**Reject**
*Maieta* monophyletic	+1	0.000	1.000	Cannot reject
*Mecranium* monophyletic	+10	−0.567	0.570	Cannot reject
*Miconia* monophyletic	+52	−2.545	0.010	**Reject**
*Ossaea* monophyletic	+14	−0.915	0.359	Cannot reject
*Pachyanthus* monophyletic	+14	−0.504	0.614	Cannot reject
*Pleiochiton* monophyletic	+1	−0.077	0.937	Cannot reject
*Sagraea* monophyletic	+5	−0.341	0.732	Cannot reject
*Tetrazygia* monophyletic	+9	−0.441	0.658	Cannot reject
*Tococa* monophyletic	+19	−0.879	0.379	Cannot reject
**CLADE CONSTRAINTS**				
Caribbean (*a*)	+8	−0.285	0.775	Cannot reject
Caribbean (*b*)	+29	−1.821	0.068	Cannot reject
Clidemia grade (*a*)	+7	−0.228	0.819	Cannot reject
Clidemia grade (*b*)	+23	−1.672	0.094	Cannot reject
Clidemia grade (*c*)	+3	−0.105	0.916	Cannot reject
Clidemia grade (*d*)	+4	−0.140	0.888	Cannot reject
Clidemia grade (*e*)	+6	−0.420	0.668	Cannot reject
Conostegia s.l.	+22	−1.195	0.231	Cannot reject
Leandra + Ossaea (scorpioid)	+3	−0.103	0.917	Cannot reject
Leandra s.s. + Pleiochiton	+24	−1.397	0.162	Cannot reject
Mecranium + Anaectocalyx + allies	+20	−0.736	0.461	Cannot reject
Miconia I	+7	−0.026	0.979	Cannot reject
Miconia II	+5	−0.526	0.598	Cannot reject
Miconia III	+30	−1.540	0.123	Cannot reject
Miconia IV	+33	−1.854	0.063	Cannot reject
Miconia V grade (*a*)	+5	−0.286	0.774	Cannot reject
Miconia V grade (*b*)	+24	−1.376	0.168	Cannot reject
Miconia V grade (*c*)	+6	−0.627	0.530	Cannot reject
Ossaea p.p.	+5	−0.547	0.584	Cannot reject
Tococa s.s.	+10	−0.214	0.830	Cannot reject

### Ancestral character reconstruction and phylogenetic signal

**Figure 3:**
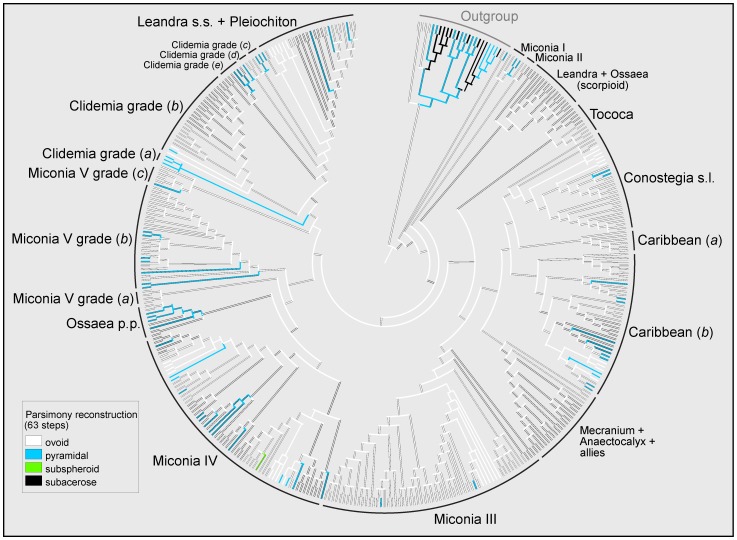
Ancestral character reconstruction of seed shape in Miconieae. Tree with the highest likelihood from the Bayesian run derived from the analysis of a combined data matrix of ITS, ETS, *accD-psaI* and *psbK-psbI* DNA sequences. The ancestral character reconstruction was performed using the parsimony criterion and considering the character states as unordered. Clade names were adopted, with some modifications, from Goldenberg et al. [25].

The characters under study were homoplasious (Table 3; an example of the homoplasious nature of seed characters is shown in Figure 3, while the ancestral character reconstruction history of all traits under study is found in Figure S2). Reconstruction of the ancestral character states for the seeds of Miconieae retrieved identical results for most characters using both MP and Bayesian trees. The exceptions were the microrelief of the periclinal walls and the length of the seed from the antiraphal view, whose reconstruction was ambiguous in the MP and the Bayesian trees, respectively. Following is a description of the estimated morphology of the ancestral seed of Miconieae:

**Table 3:** Statistics for the seed morphological characters when reconstructed on the molecular phylogeny (parsimony criterion).

Character	CI	RI
Three-dimensional shape	0.38	0.3
Base of the body of the seed horizontally expanded	0.5	0.67
Location of the highest point perpendicular to the raphal zone	0.44	0.22
Symmetrical plane of the raphal zone	0.48	0.28
Length of the raphal zone proportional to the total length of the seed	0.02	0.41
Ventrally-oriented expansion of the raphal zone	0.08 (0.09)	0.21 (0.28)
Appendage	0.02	0.35
Multicellular sculpture	0.01	0.46
General arrangement of the cells with respect to each other	0.03	0.48
Cell shape	0.44	0.19
Relief of the anticlinal walls	0.14	0
Curvature of the anticlinal walls	0.6 (0.58)	0.16 (0.10)
Relief of the periclinal walls	0.04	0.39
Periclinal walls dividing into two or more segments	0.03	0.22
Microrelief of the periclinal walls	0.01	0.39
Cells with features differing from the rest of the seed corpus	0.04	0.17
Length of the seed from antiraphal view (mm)	0.5	0.39

If the values obtained for one of the most parsimonious trees (maximum parsimony) and the tree with the highest likelihood (Bayesian analysis) were different, the value for the Bayesian tree is shown within parentheses. CI =  Ensemble consistency index; RI =  Ensemble retention index.

Seed ovoid, without the base of the body of the seed horizontally expanded, <0.5 mm (MP tree) or <1 mm long (Bayesian tree); the highest point towards the chalazal side. Raphal zone ovate, 70–85% the length of the seed, not ventrally expanded. Appendage absent. Multicellular sculpture present. Cells arranged in an irregular pattern. Cells of the seed corpus homogeneous; individual cells elongated, anticlinal boundaries channeled, undulate; periclinal walls flat to convex, without dividing into several segments, microrelief absent (ambiguous in the MP tree).

This combination of ancestral character states was found in 11 species of the “Conostegia s.l.”, “Mecranium + Anaectocalyx + allies”, “Miconia III”, “Miconia IV”, and “Miconia V grade (*b*)” clades (Table S2). MacClade could not detect any seed morphological synapomorphies within Miconieae, and only the subspheroid shape of the seed of *Miconia chartacea* was found as an autapomorphy (results not shown).

Although seed morphology seems to be homoplasious, the analyses detected that the distribution of character states in the phylogenies was significantly different from that expected by chance (Table 4). All characters had a significant value of *P*<0.01 except the curvature of the anticlinal walls which retrieved a marginally significant value when evaluated in the Bayesian tree (*P* = 0.0495). Only the relief of the anticlinal walls did not show better structured data than the randomly permuted data set.

**Table 4:** Probability that the distribution of the seed morphological characters is random with respect to the phylogeny estimated with the DNA sequence data.

	Parsimony tree			Bayesian tree		
Character	Number of steps in the reference tree	Number of trees with equal or lower number of steps than the reference tree	*P*	Number of steps in the reference tree	Number of trees with equal or lower number of steps than the reference tree	*P*
Three-dimensional shape	63	0	0	63	0	0
Base of the body of the seed horizontally expanded	2	1	0.0001	2	0	0
Location of the highest point perpendicular to the raphal zone	86	1	0.0001	86	1	0.0001
Symmetrical plane of the raphal zone	138	0	0	138	0	0
Length of the raphal zone proportional to the total length of the seed	119	0	0	118	0	0
Ventrally-oriented expansion of the raphal zone	12	6	0.0006	11	1	0.0001
Appendage	48	0	0	48	0	0
Multicellular sculpture	86	0	0	86	0	0
General arrangement of the cells with respect to each other	36	0	0	36	0	0
Cell shape	61	5	0.0005	61	5	0.0005
Relief of the anticlinal walls	21	10,000	1	21	10,000	1
Curvature of the anticlinal walls	40	56	0.0056	41	495	0.0495
Relief of the periclinal walls	51	0	0	51	0	0
Periclinal walls dividing into two or more segments	29	0	0	29	0	0
Microrelief of the periclinal walls	67	0	0	67	0	0
Cells with features differing from the rest of the seed corpus	26	10	0.001	26	13	0.0013
Length of the seed from antiraphal view (mm)	126	0	0	128	0	0

### Distribution of morphological seed types in the phylogeny

Figure S1 shows the distribution of the morphotypes proposed in previous studies in a Bayesian 50% majority-rule consensus tree. Our sampling includes 20 taxa that represent all the supertypes and types proposed by Groenendijk et al. [22] (except the Centrodesma, Mesmeana, and Ternatifolia types), and 47 species that correspond to all the types described by Martin and Michelangeli [33] (except their type IIb). Although the number of species with morphotype ascription was low, it was enough to show that the types in Groenendijk et al. [22] were distributed in five clades, each of which included more than one type. This was especially evident in the Miconia III clade (four supertypes and seven types). On the other hand, the distribution of the morphological types in the phylogeny proposed by Martin and Michelangeli [33] suggested that some of them could be associated with specific clades; however, the morphotype assignment of 22 additional samples showed the same pattern as in the previous case, except that the “Leandra s.s. + Pleiochiton” clade included four morphotypes and six subtypes. The “Clidemia grade (*c*)” clade had species with only type I seeds, although that morphotype can also be found in the “Miconia V grade (*c*)” clade (*Clidemia involucrata*). For a summary of the distribution of the seed morphological types in the major clades of the phylogeny see Tables 5 and 6.

**Table 5:** Distribution of seed morphological types sensu Groenendijk et al. [22] in the molecular phylogeny.

Supertype	Type	Type name	Clade
I	1	Buxifolia	Mecranium + Anaectocalyx + allies
			Miconia III
	2	Notabilis	Miconia III
	3	Affinis	Miconia IV
			Miconia V grade b
	4	Ternatifolia	*
II	5	Chionophylla	Miconia III
	6	Mesmeana	*
	7	Centrodesma	*
III	8	Reducens	Miconia III
			Miconia V grade a
	9	Benthamiana	Miconia III
	10	Lacera	Mecranium + Anaectocalyx + allies
			Miconia III
IV	11	Traillii	Miconia IV
			Miconia V grade a
V	12	Tomentosa	Miconia V grade a
VI	13	Miscellaneous	Miconia III
			Miconia IV
			Miconia V grade b

*Seed type not included in our sampling.

**Table 6:** Distribution of seed morphological types sensu Martin and Michelangeli [33] in the molecular phylogeny.

Type	Subtype (if applicable)	Clade
I		Clidemia grade c
		Miconia V grade c
II	a	Clidemia grade d
		Miconia IV
	b	*
III		Caribbean b
		Mecranium + Anaectocalyx + allies
IV		Ossaea p.p.
V		Leandra s.s. + Pleiochiton
		Miconia IV
VI	a	Clidemia grade a
		Leandra s.s. + Pleiochiton
	b	Leandra s.s. + Pleiochiton
		Miconia III
VII		Ossaea p.p.
VIII	a	Leandra s.s. + Pleiochiton
	b	Leandra s.s. + Pleiochiton
	c	Caribbean b
		Leandra s.s. + Pleiochiton
	d	Leandra s.s. + Pleiochiton
IX		Clidemia grade b
		Leandra + Ossaea (scorpioid)
X		Clidemia grade b
		Conostegia s.l.
		Mecranium + Anaectocalyx + allies
XI		Caribbean b
		Clidemia grade a
		Clidemia grade d
XII	a	Miconia II
		Miconia V grade c
	b	Miconia II
XIII		Leandra s.s. + Pleiochiton
XIV		Leandra + Ossaea (scorpioid)
		Mecranium + Anaectocalyx + allies
XV		Miconia III
XVI		Clidemia grade b

*Seed type not included in our sampling.

## Discussion

In this study, we evaluated in a phylogenetic context the taxonomic, systematic, and evolutionary implications of seed morphology diversity of ca. 20% of the species of Miconieae. Previous studies showed that Miconieae seeds show extensive morphological variation and that they may be useful for revealing evolutionary patterns [22], [26], [33] as suggested for other groups in the Melastomataceae [19]–[21]. Although our results confirm significant seed morphological variation in the tribe, the analyses show that seed characters are highly homoplasious and that their use for circumscribing monophyletic genera and higher taxonomic groups is rather limited.

### Morphotypes and composite coding

A common feature found in previous investigations is the creation of multiple morphological types for describing seed diversity in the Melastomataceae [20], [22], [33], [57]. These morphological types are not directly comparable among studies and they are usually named after a species [20], [22], or are assigned arbitrary numbers [33]. The comparison of morphological types from different studies is fraught with difficulties because each type is a collection of character states, and sometimes the characters and/or terminology for describing them is not consistent among investigations. The creation of morphological types is known as composite coding [34], an approach that incorporates the variation of several characters into a single character state. Morphological types may be useful for summarizing the overall morphological diversity of a particular feature, but it is also a procedure that may render undesired results. For instance, complex classification systems are developed in order to accommodate the whole morphological variation and may cause potential confusion: subcategories are created to describe slight deviations from the general pattern (see [33]) and special categories are defined for those samples with heterogeneous morphologies that cannot be associated with other proposed types (see [22], [58]). In addition, this approach has been criticized because it does not take advantage of the entire range of variation for individual characters, may create putative synapomorphies not present in them, and can mislead phylogenetic inference [59]. Because of these potential problems, we opted for analyzing individual characters instead of morphological types, and used a subset of the characters proposed by Ocampo and Almeda [31]. Because of the wide range of morphological variation found among and within species, some of the characters were coded as polymorphic. The inclusion of polymorphic data in phylogenetic and evolutionary analyses has been controversial, and while some authors argue that they are not reliable for estimating evolutionary relationships [60], [61], other studies show that inclusion of polymorphic data is important because they may contain phylogenetic information [62], [63].

### Taxonomic implications

It is well known that the genera within Miconieae are, morphologically speaking, poorly characterized [18], [27]–[30]. It is not uncommon for the morphological character states traditionally employed for distinguishing genera to overlap. This makes the generic assignment of some species ambiguous. The seed morphological features are no exception. Although they are diverse, they provide no magic bullet for the assignment of species to genera which is in agreement with previous investigations focused on the Miconieae [22], [26]. The phylogenetic analyses of morphological data retrieved a topology with very low resolution, and the few recovered clades do not represent monophyletic genera. In addition, the hypothesis testing procedures reject the monophyly of the three largest genera which collectively comprise over 80% of the species in the Miconieae (*Clidemia*, *Leandra* and *Miconia*, although the first one presents a marginal value of *P* = 0.05). Seed morphology in the Melastomataceae has been used to some extent for characterizing a number of capsular-fruited tribes [20]. Seed morphology has been shown to be of value in characterizing monophyletic genera like *Siphanthera* [64] but its widespread utility for circumscribing monophyletic capsular-fruited genera remains to be demonstrated. While Baumgratz [21] proposed that seed morphology could be used to cluster some Brazilian genera (and occasionally discriminate a number of them), other studies show that seed characters of Miconieae species do not support generic circumscription or subgeneric classifications of *Leandra* [26], *Miconia* [22], [31], and *Tococa* [32]. Although seed features are variable and overlap among traditionally recognized genera of Miconieae [22], [31], there is evidence that some seed characters in concert with other vegetative and reproductive traits may support a limited number of monophyletic groups (see below).

### Systematic and evolutionary implications

The lack of a strict association between seed morphology and traditional classifications not only questioned the circumscription of some taxa, but at the same time suggested that seed features may be helpful for estimating phylogenetic relationships within Melastomataceae [19]–[22]. This assumption is based on the idea that seed characters, unlike floral features, represent a conserved trait and, consequently, can inform the evolutionary history of flowering plants [2], [20]. Although hypothesis testing procedures cannot reject the monophyly of the main clades proposed in Michelangeli et al. [23] and Goldenberg et al. [25], our study shows that seed characters are homoplasious and provide limited information for estimating evolutionary relationships within the Miconieae. It is noteworthy that the Bayesian analysis of the morphological data recovered six pairs of species that are consistent with the relationships obtained by the analyses of DNA sequence data [23], [25], and in this study; in addition, the MP analysis partially recovered the “Leandra + Ossaea (scorpioid)” clade. Although these relationships have very low BS and p.p. values, the fact that they are recovered in the phylogenetic analyses suggest that seed features could support relationships among a limited number of taxa. Groenendijk et al. [22] speculated that seed features could be an important source of data for recovering phylogenetic relationships within the Miconieae. Although our sampling does not include all the morphological types described by those authors, the distribution of the morphotypes in our molecular phylogeny clearly shows that they occur in several places and none of them seems to be associated with a specific clade.

On the other hand, Martin et al. [26] showed that a specific combination of seed characters could be associated with 10 clades within Miconieae, although their conclusions were based on the morphological analysis of ca. 25% (mainly *Leandra* species) of the samples used for estimating their molecular phylogeny. Our study, which increased taxon sampling for both morphological and molecular data, included representatives of all but one of the morphotypes proposed by Martin and Michelangeli [33]. Our data shows that their morphological types are not specific to any clade since they can be found in different parts of the phylogeny. We found it very difficult to unambiguously assign morphotypes to our species samples, in spite of the claim that this seed classification easily separates seed diversity into discrete groups [33]. This difficulty is probably a reflection of our expanded sampling of Miconieae which includes greater diversity than previously described and the inclusion of potentially undescribed morphotypes. This is particularly evident in the shape and length of the seed, relative length of the raphal zone, and relief and microrelief of the periclinal walls. In addition, the morphological type circumscriptions in Martin and Michelangeli [33] include many exceptions. Discrepancies throughout their study also made it difficult to unambiguously identify the seed morphological type for some of our samples. We encountered difficulties using their dichotomous key to determine types X and XVI coupled with inconsistent designations of types XV and XVI. One way to avoid these problems would be to refine seed type circumscriptions and to create more morphotypes or subtypes, although that would likely cause a more convoluted classification system [31] and the use of composite coding could negatively impact phylogenetic inferences [59].

The individual seed traits that are considered in this study are homoplastic and do not appear to be associated with any particular clade. Michelangeli [32] showed that the straight anticlinal walls (coded as irregularly curved in our study) were a synapomorphy for *Tococa s.s*.; however, increased taxon sampling shows that this character evolved multiple times in the Miconieae. Likewise, suites of morphological character states seem to have converged in independent lineages. This is the case for the states estimated for the ancestral Miconieae seed, which have evolved at least 11 times in five different clades. Although the seed features do not seem to be diagnostic for specific clades, it is apparent that seed morphology can be coupled with other vegetative and reproductive characters to identify some groups. To be sure, seed characters are of value in circumscribing species as noted previously [31]. For instance, the presence of simple (eglandular or glandular) hairs, flowers arranged in a cymose helicoid pattern, and ovoid seeds with aligned cells and par-convex periclinal walls are typical of the members of the “Leandra + Ossaea (scorpioid)” clade [65], except *Ossaea capillaris*, which has convex periclinal walls. We could not identify other combinations of character states in this study that could be associated with the specific clades identified by Goldenberg et al. [25]. However, we suspect that a potential set of characters could identify smaller groups, as already suggested in other studies [32], [66]. For example, a subclade of the “Conostegia s.l.” clade can be characterized by the presence of a calyptra and seeds with the base of the body horizontally expanded (Ricardo Kriebel, unpublished results); the clade formed by the species of *Pleiochiton* [67] (“Leandra s.s. + Pleiochiton” clade) can be diagnosed by the epiphytic habit, succulent roots, and seeds with aligned cells; similarly, the presence of papillose petals, seeds with punctate microrelief, and an expanded raphal zone larger than the seed body seem to be a characteristic of a small group within the “Ossaea p.p.” clade [68] (“Quinquenervia” suclade of the “Octopleura” clade of *Miconia*).

Although the seed characters were shown to be labile and to have evolved multiple times within Miconieae, almost all of them showed a significant phylogenetic signal. A strong phylogenetic signal has been associated with low rates of evolution [69]–[71], which results in a higher resemblance between related species. The estimation of phylogenetic signal may be affected by the limitations of the existing methods, the inaccuracy of the phylogenetic estimate, or by errors in the data itself [72], which may explain the apparent inconsistency of our results that show phylogenetic signal using homoplasious characters. Other factors that may affect this interpretation include errors in the characterization and coding of the character states [73]. However, other authors consider that phylogenetic signal may not be associated with evolutionary rates, and recommend that phylogenetic signal should not be used to make interpretations about evolutionary processes [74]. Homoplasious, continuous characters under a Brownian motion model of evolution have been associated with adaptive evolution [72]. Although the seed traits were studied under a randomization procedure without an evolutionary model because of the nature of the data (discrete polymorphic characters), we cannot discard the possibility that diversification of seed characters has an adaptive component as suggested by some studies [75], [76]. Groenendijk et al. [22] concluded that some seed features may enhance secondary dispersal by ants and noted that some seed types were more abundant at certain elevational ranges, suggesting that seed morphology was selected by ecological and environmental pressures. Preliminary results show that there is a positive correlation between multicellular sculpture and precipitation (Gilberto Ocampo and Frank Almeda, unpublished results), which may decrease wettability [77], [78] and enhance seed flotation for short-distance secondary dispersal by water. There is evidence that seed traits may be impacted by environmental variables, but there are still many questions to be addressed in order to understand the forces that drive seed morphological evolution, as well as the complex molecular mechanisms that control those changes [79]. Also, seed size may be impacted by other factors such as polyembryonic seeds in apomictic species [80] and selective pressures that favor seed longevity [81], [82].

## Conclusions

Our results show that although seed morphological characters are very variable and diverse, they alone do not unequivocally support the circumscription of genera and clades. However, expanded sampling and character evaluation in the tribe can supply further information to characterize smaller clades. For instance, it is known that a group within the “Tococa s.s.” clade has glandular hairs on the raphal zone [32], a unique character within Miconieae that was not evaluated here, nor was it evaluated in [31]. Similarly, description of seed shape by geometric morphometric methods seems to be more accurate than categorical coding (Ricardo Kriebel and Gilberto Ocampo, unpublished results), and further anatomical evaluations may provide a more objective characterization of seed appendages (Rafaella Ribeiro, unpublished data). Finally, more studies are needed to interpret the putative relationships among seed morphological features and selective pressures, so we can obtain further insights into the forces that drive seed morphological diversification in the tribe Miconieae.

## Supporting Information

Figure S1 **Bayesian 50% majority-rule consensus tree showing a representative scanning electron microscopy image of the seed for each species (images not to scale).** The relationships were estimated from the analysis of a combined data matrix of ETS, ITS, *accD-psaI* and *psbK-psbI* DNA sequences. Posterior probabilities are indicated at each node. Outgroup taxa not shown. Clade names were adopted, with some modifications, from Goldenberg et al. [25]. The morphological type ascription sensu Groenendijk et al. [22] and Martin and Michelangeli [33] is indicated for some species. Putative morphological type ascription of some species (determined for this study) is indicated in underlined text. * =  as assigned in the description of Martin and Michelangeli [33]; ** =  seed sample does not perfectly match the seed morphological description of Martin and Michelangeli [33].
(PDF)

Figure S2 **Ancestral character reconstruction of the 17 seed morphological characters used in this study.** One of the most parsimonious trees (left) and the tree with the highest likelihood from the Bayesian run (right) derived from the analysis of a combined data matrix of ITS, ETS, *accD-psaI* and *psbK-psbI* DNA sequences are shown. Ancestral character reconstruction was performed using the parsimony criterion and considering the character states as unordered. Clade names were adopted, with some modifications, from Goldenberg et al. [25].
(PDF)

Table S1 Species name, source from which seed information was obtained (voucher information of plant material or literature reference), and GenBank accession numbers (ETS, ITS, *accD-psaI*, and *psbK-psbI*). Taxa are arranged in alphabetical order by genus and species. Outgroup taxa are shown at the end of the table. NA =  not available; * =  SEM image taken at CAS; ‡ = SEM image taken at NY.
(PDF)

Table S2 Morphological data matrix. Numbers correspond to characters and character states shown in the Materials and Methods section. Notes: * =  character with polymorphic data; ? =  missing data; ‡ =  species with a combination of character states that were estimated for the ancestral Miconieae seed; § =  outgroup taxa.
(PDF)
