# Supplementary material for: Seed Diversity in the Tribe Miconieae (Melastomataceae): Taxonomic, Systematic, and Evolutionary Implications
Source: PLoS One. 2014 Jun 23;9(6):e100561. doi: 10.1371/journal.pone.0100561 (PMC4067357; doi:10.1371/journal.pone.0100561)
Supplement: Figure S1 — Bayesian 50% majority-rule consensus tree showing a representative scanning electron microscopy image of the seed for each species (images not to scale). The relationships were estimated from the analysis of a combined data matrix of ETS, ITS, accD-psaI and psbK-psbI DNA sequences. Posterior probabilities are indicated at each node. Outgroup taxa not shown. Clade names were adopted, with some modifications, from Goldenberg et al. [25]. The morphological type ascription sensu Groenendijk et al. [22] and Martin and Michelangeli [33] is indicated for some species. Putative morphological type ascription of some species (determined for this study) is indicated in underlined text. * = as assigned in the description of Martin and Michelangeli [33]; ** = seed sample does not perfectly match the seed morphological description of Martin and Michelangeli [33]. (PDF) [file pone.0100561.s001.pdf]

Figure S1. Bayesian 50% majority-rule consensus tree showing a representative scanning electron microscopy image of the seed for each species (images not to scale). The relationships were estimated from the analysis of a combined data matrix of ETS, ITS, *accD-psaI* and *psbK-psbI* DNA sequences. Posterior probabilities are indicated at each node. Outgroup taxa not shown. Clade names were adopted, with some modifications, from Goldenberg et al. [25]. The morphological type ascription sensu Groenendijk et al. [22] and Martin and Michelangeli [33] is indicated for some species. Putative morphological type ascription of some species (determined for this study) is indicated in underlined text. * = as assigned in the description of Martin and Michelangeli [33]; ** = seed sample does not perfectly match the seed morphological description of Martin and Michelangeli [33].

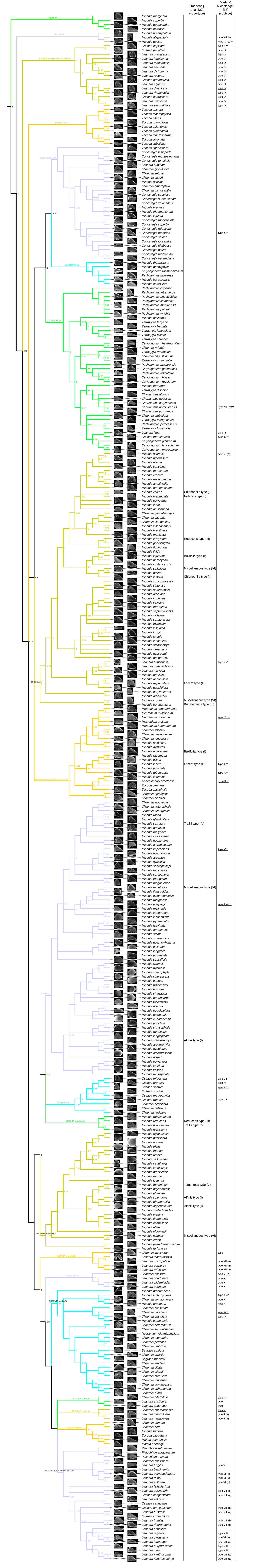
Groenendijk et al. [22] (supertype)
Martin & Michelangeli [33] (subtype)
