# Supplementary material for: Seed Diversity in the Tribe Miconieae (Melastomataceae): Taxonomic, Systematic, and Evolutionary Implications
Source: PLoS One. 2014 Jun 23;9(6):e100561. doi: 10.1371/journal.pone.0100561 (PMC4067357; doi:10.1371/journal.pone.0100561)
Supplement: Figure S2 — Ancestral character reconstruction of the 17 seed morphological characters used in this study. One of the most parsimonious trees (left) and the tree with the highest likelihood from the Bayesian run (right) derived from the analysis of a combined data matrix of ITS, ETS, accD-psaI and psbK-psbI DNA sequences are shown. Ancestral character reconstruction was performed using the parsimony criterion and considering the character states as unordered. Clade names were adopted, with some modifications, from Goldenberg et al. [25]. (PDF) [file pone.0100561.s002.pdf]

Figure S2. Ancestral character reconstruction of the 17 seed morphological characters used in this study. One of the most parsimonious trees (left) and the tree with the highest likelihood from the Bayesian run (right) derived from the analysis of a combined data matrix of ITS, ETS, *accD-psaI* and *psbK-psbI* DNA sequences are shown. Ancestral character reconstruction was performed using the parsimony criterion and considering the character states as unordered. Clade names were adopted, with some modifications, from Goldenberg et al. [25].

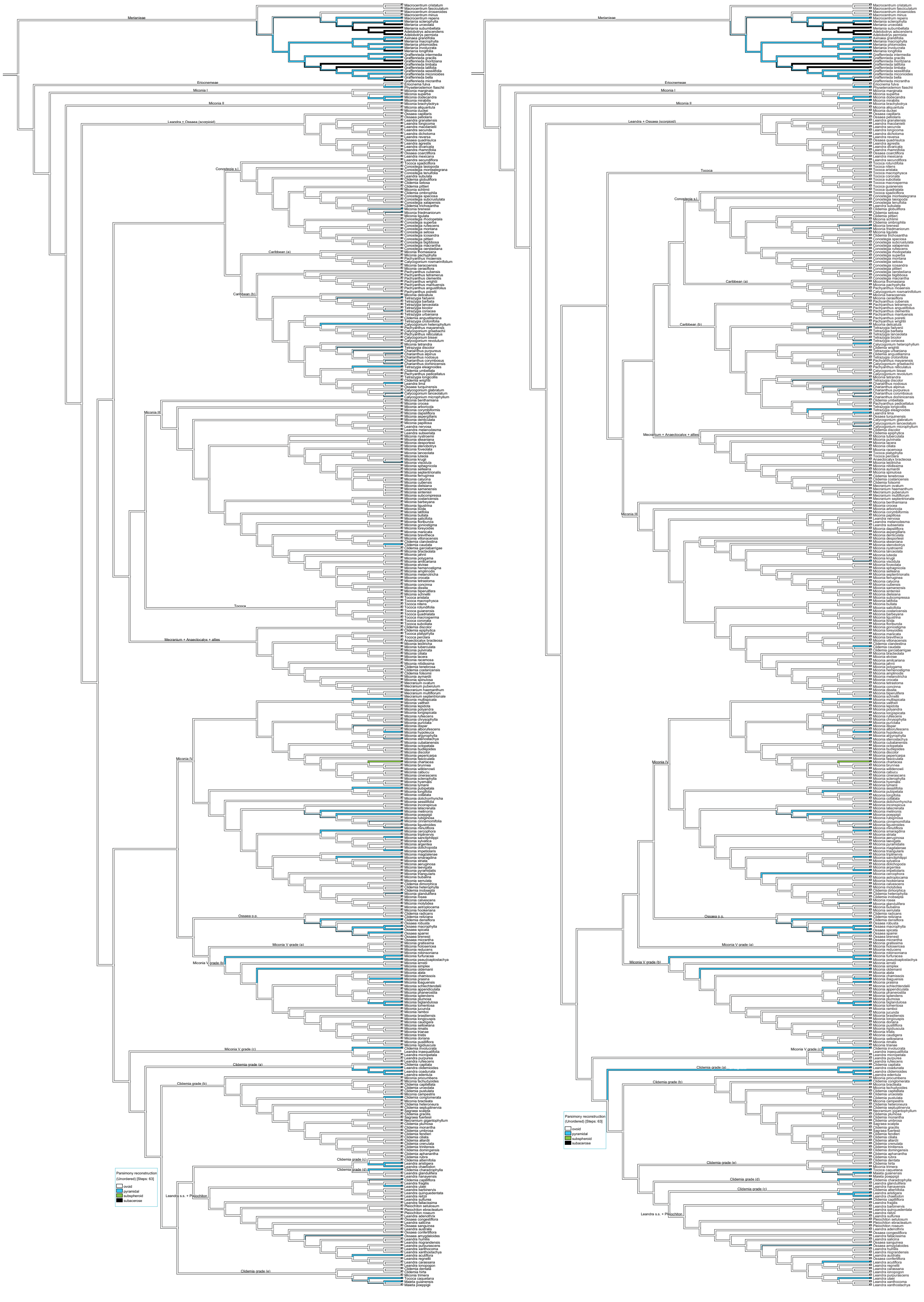

Character A. Three-dimensional shape

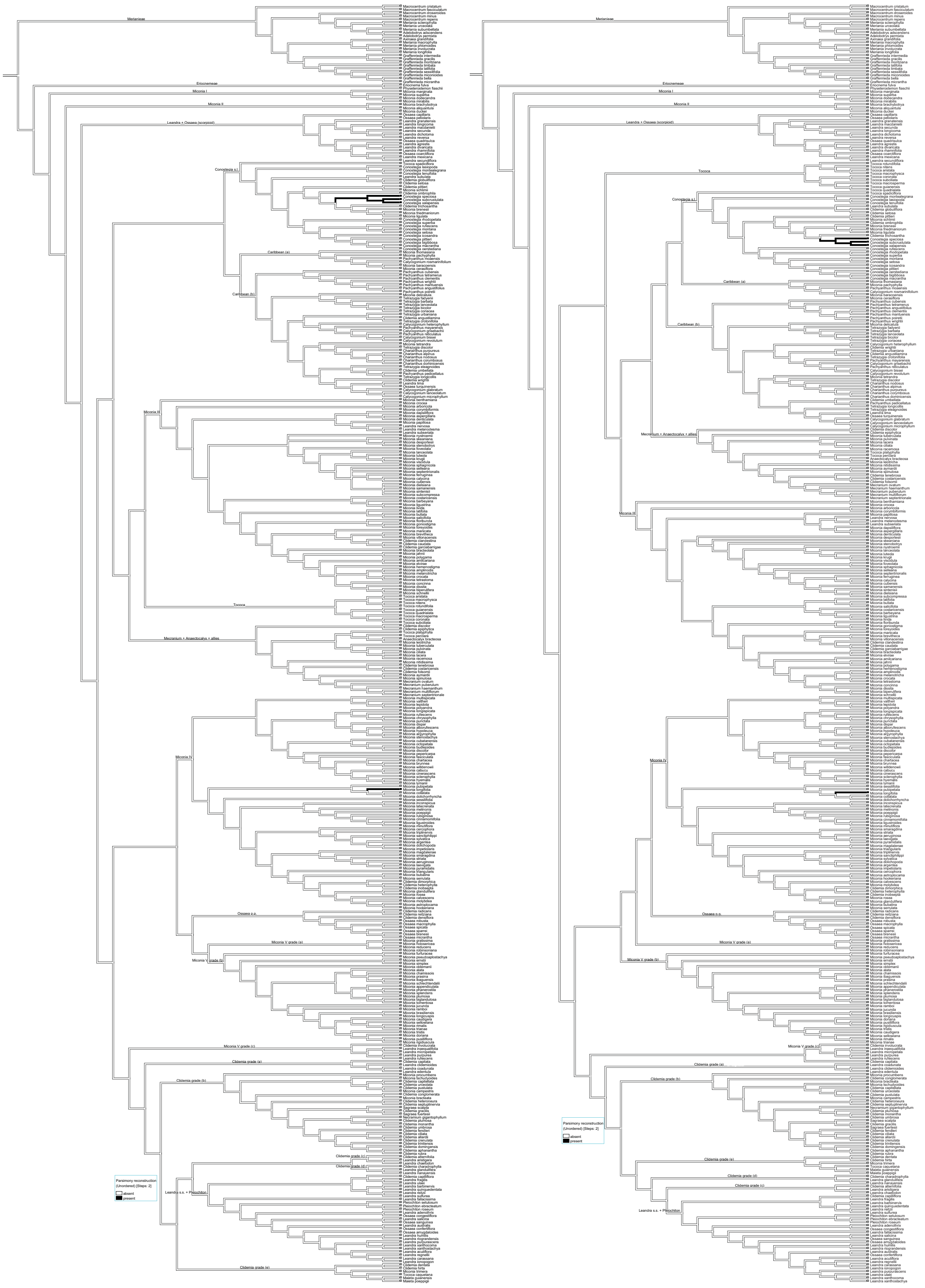

Character B. Base of the body of the seed horizontally expanded.

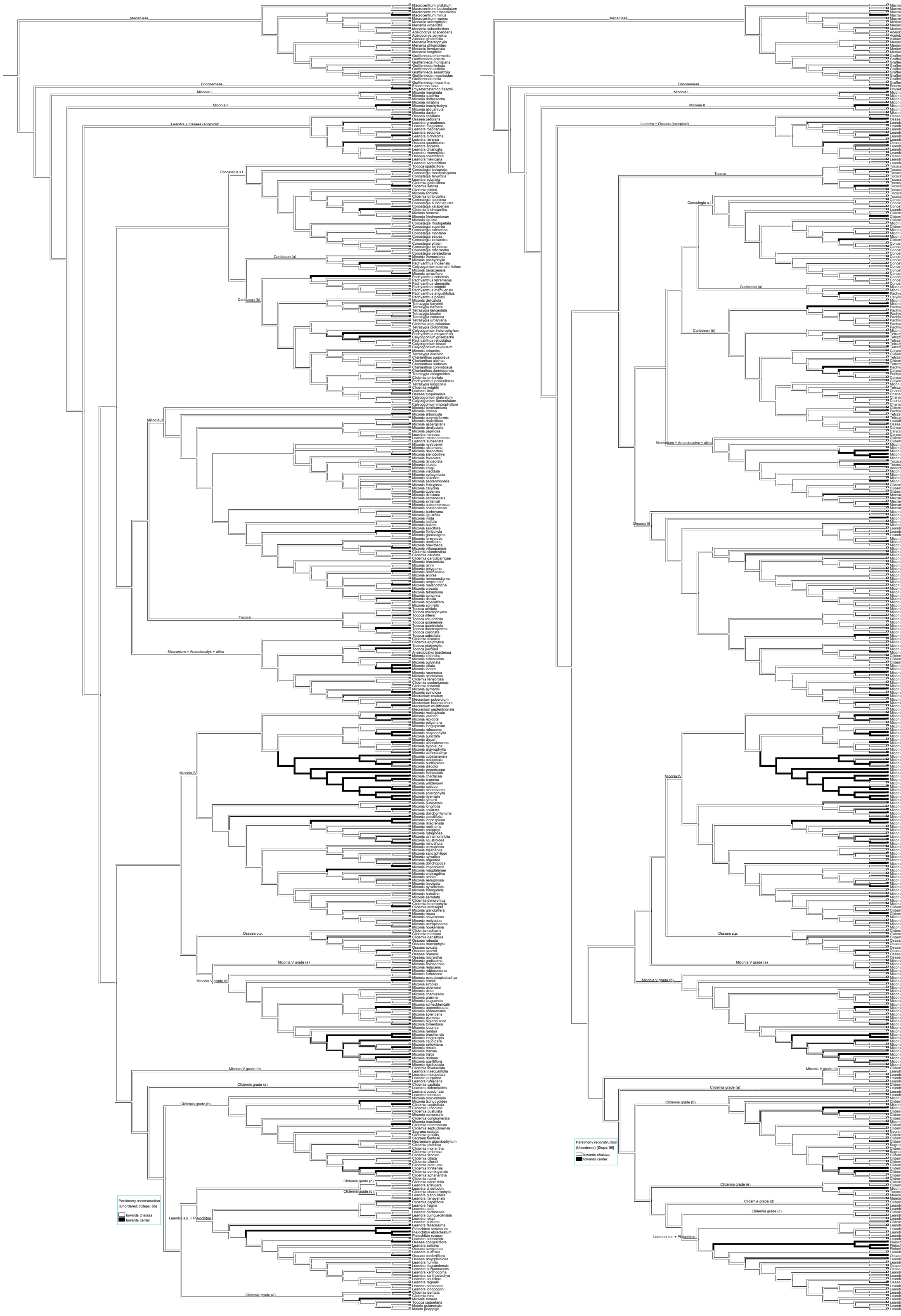

Character C. Location of the highest point perpendicular to the raphal zone.

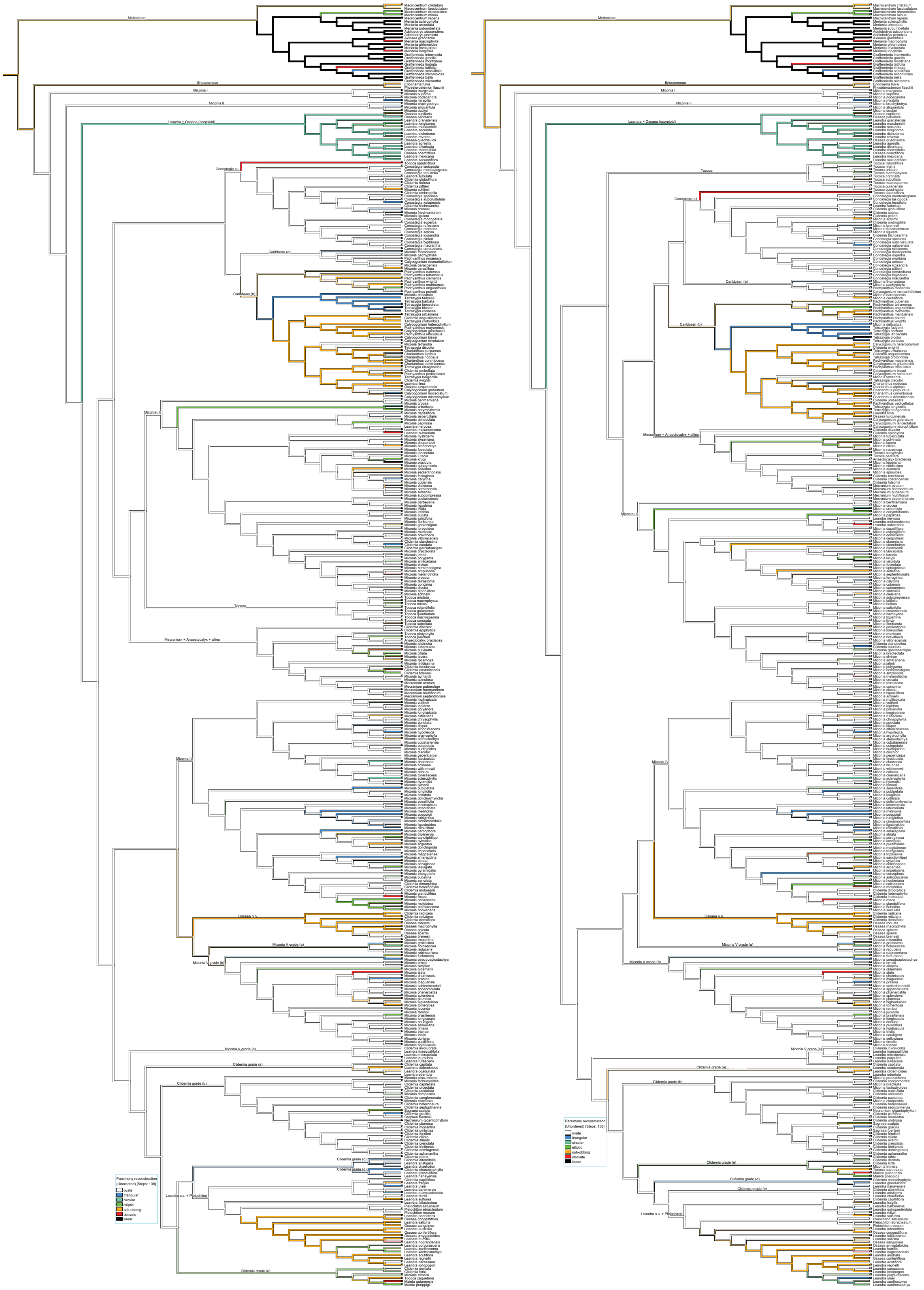

Character D. Symmetrical plane of the raphal zone.

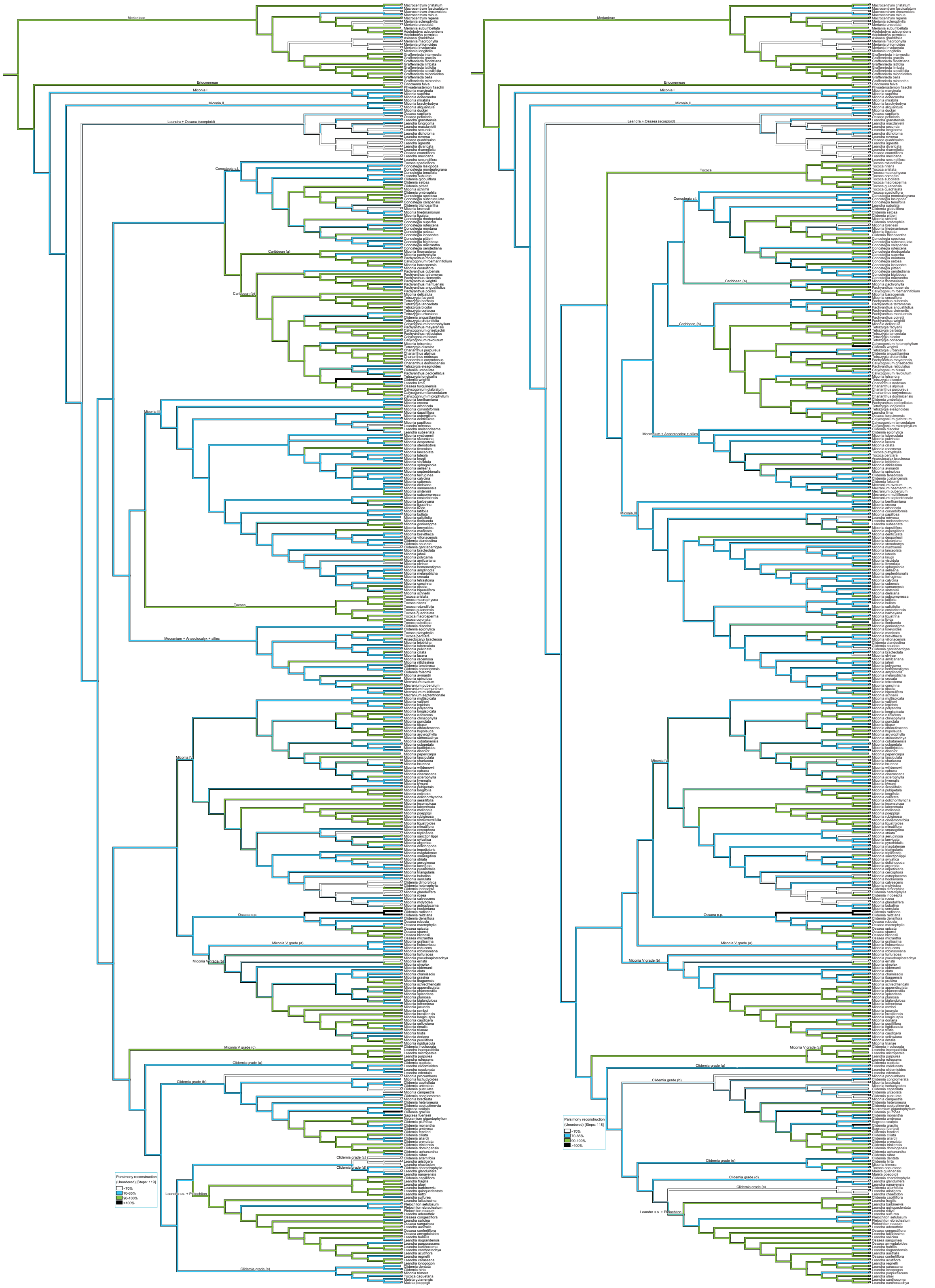

Character E. Length of the raphal zone proportional to the total length of the seed.

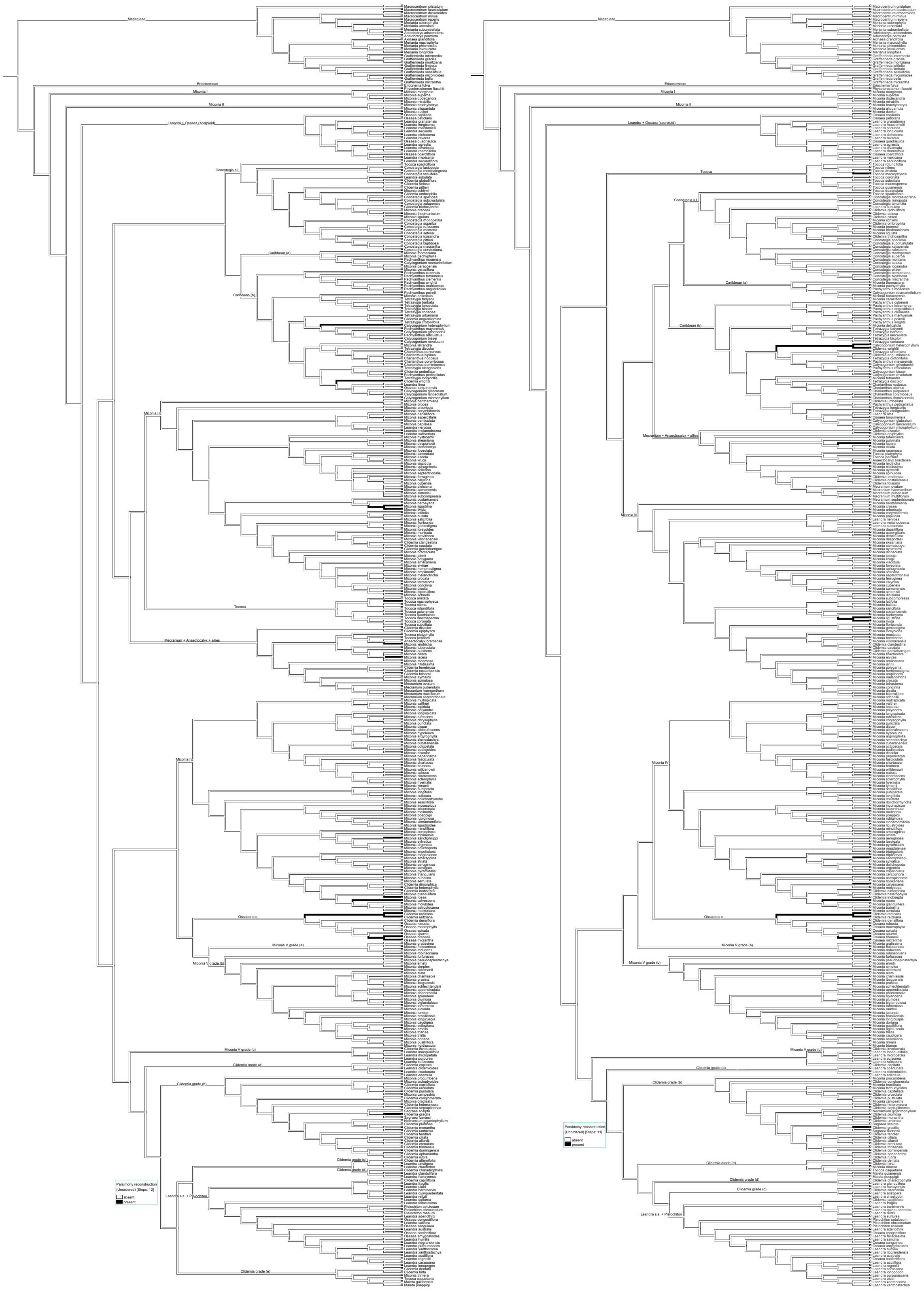

Character F. Ventrally-oriented expansion of the raphal zone.

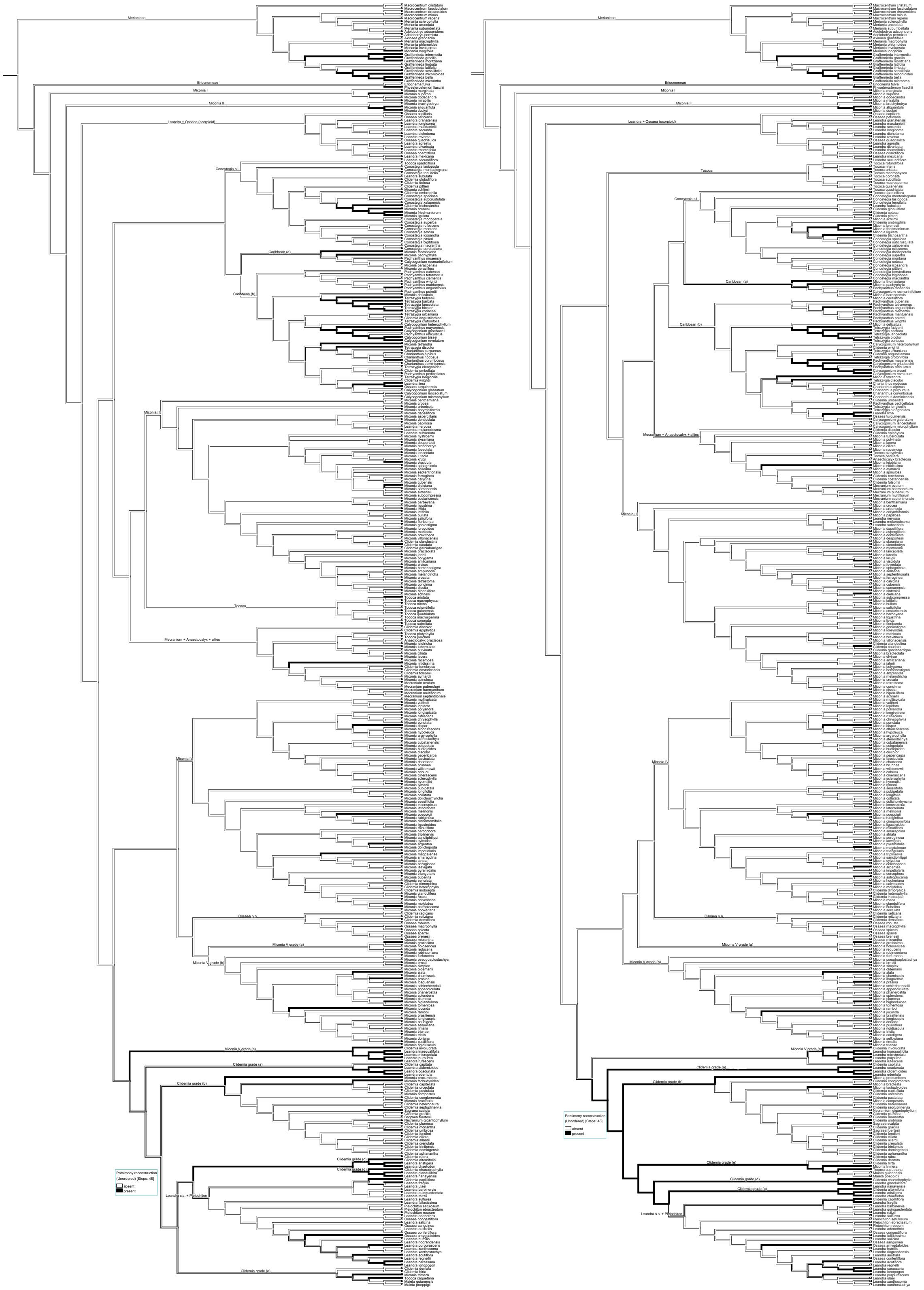

Chararacter G. Appendage.

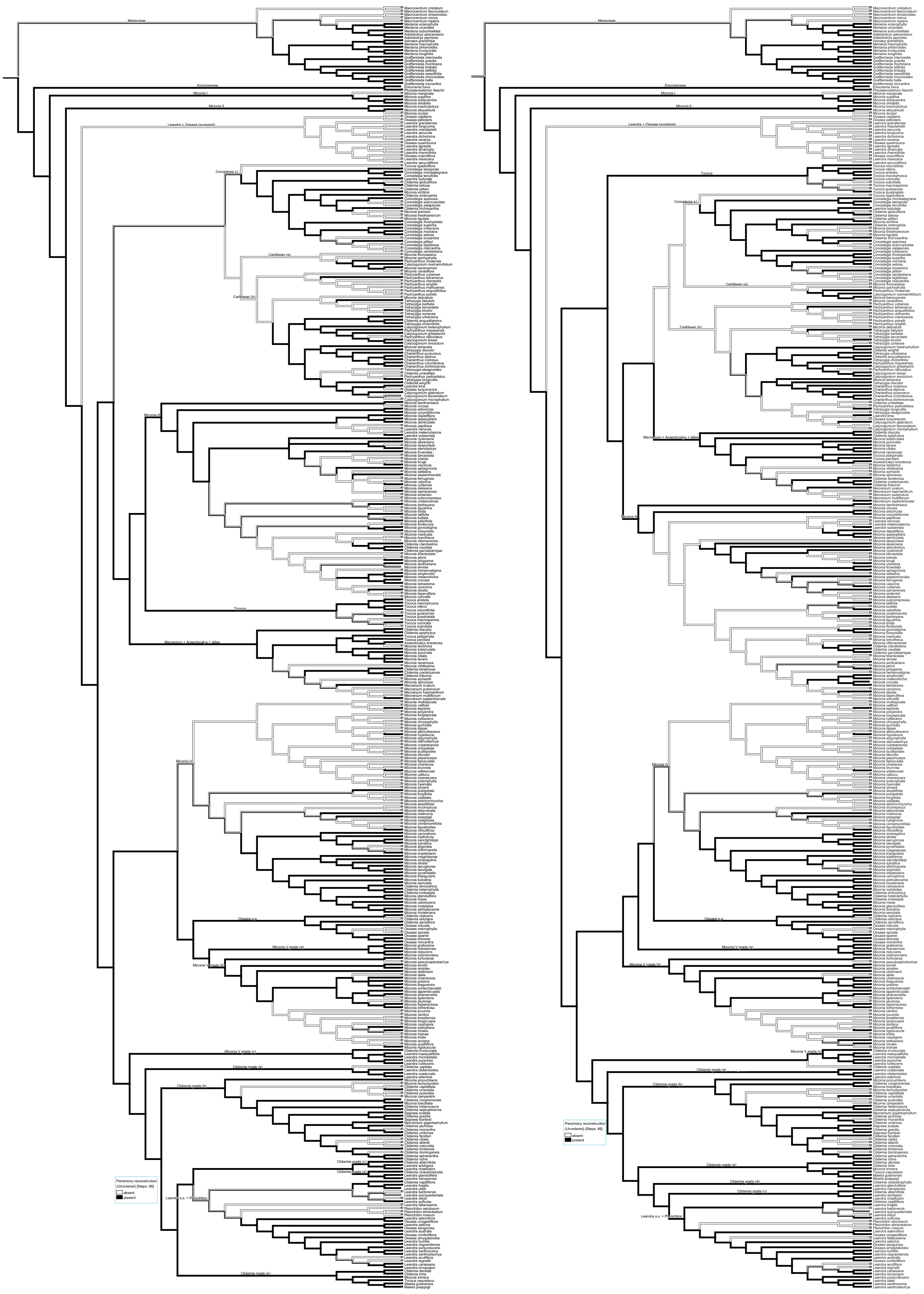

Character H. Multicellular sculpture.

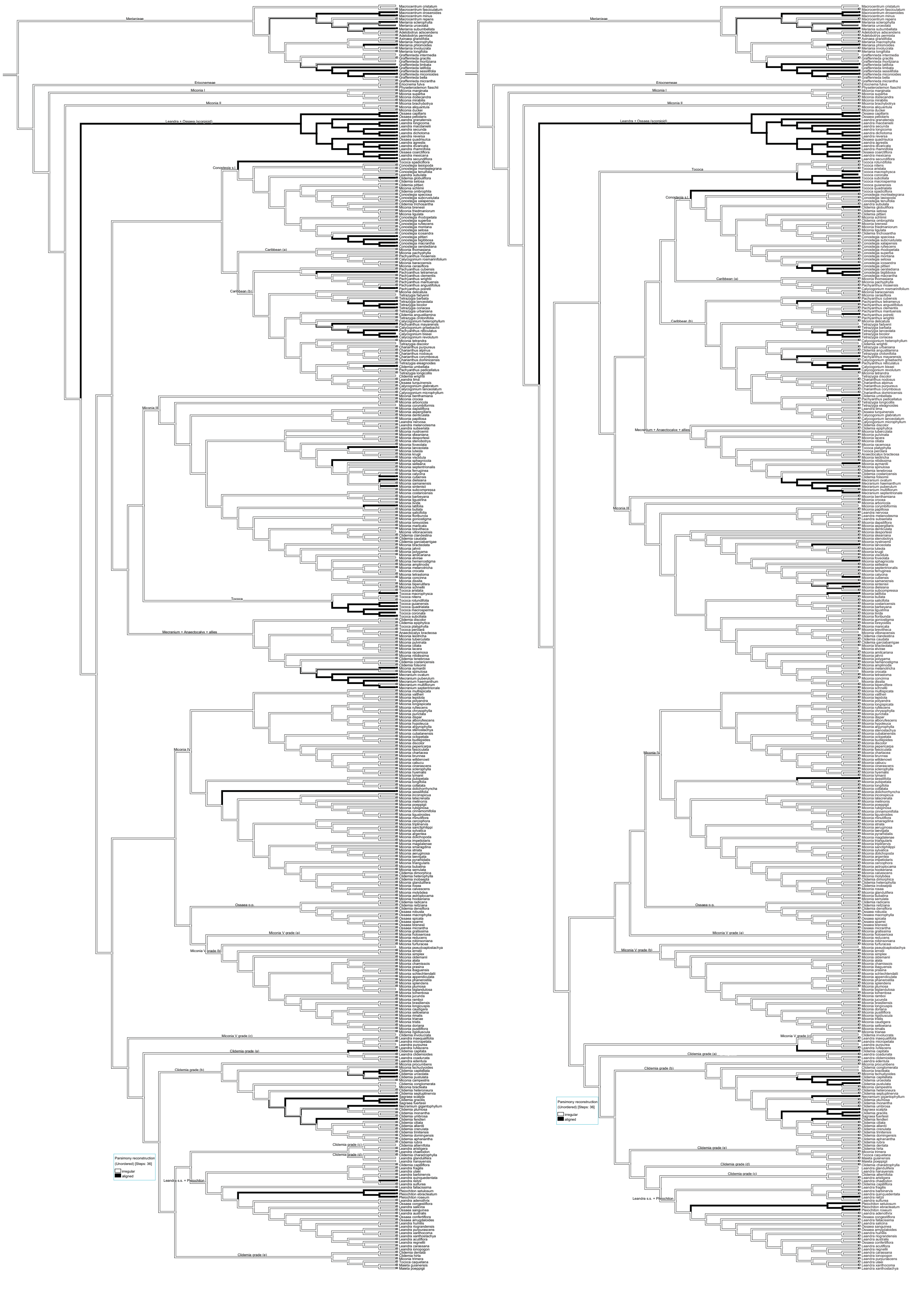

Character I. General arrangement of the cells with respect to each other.

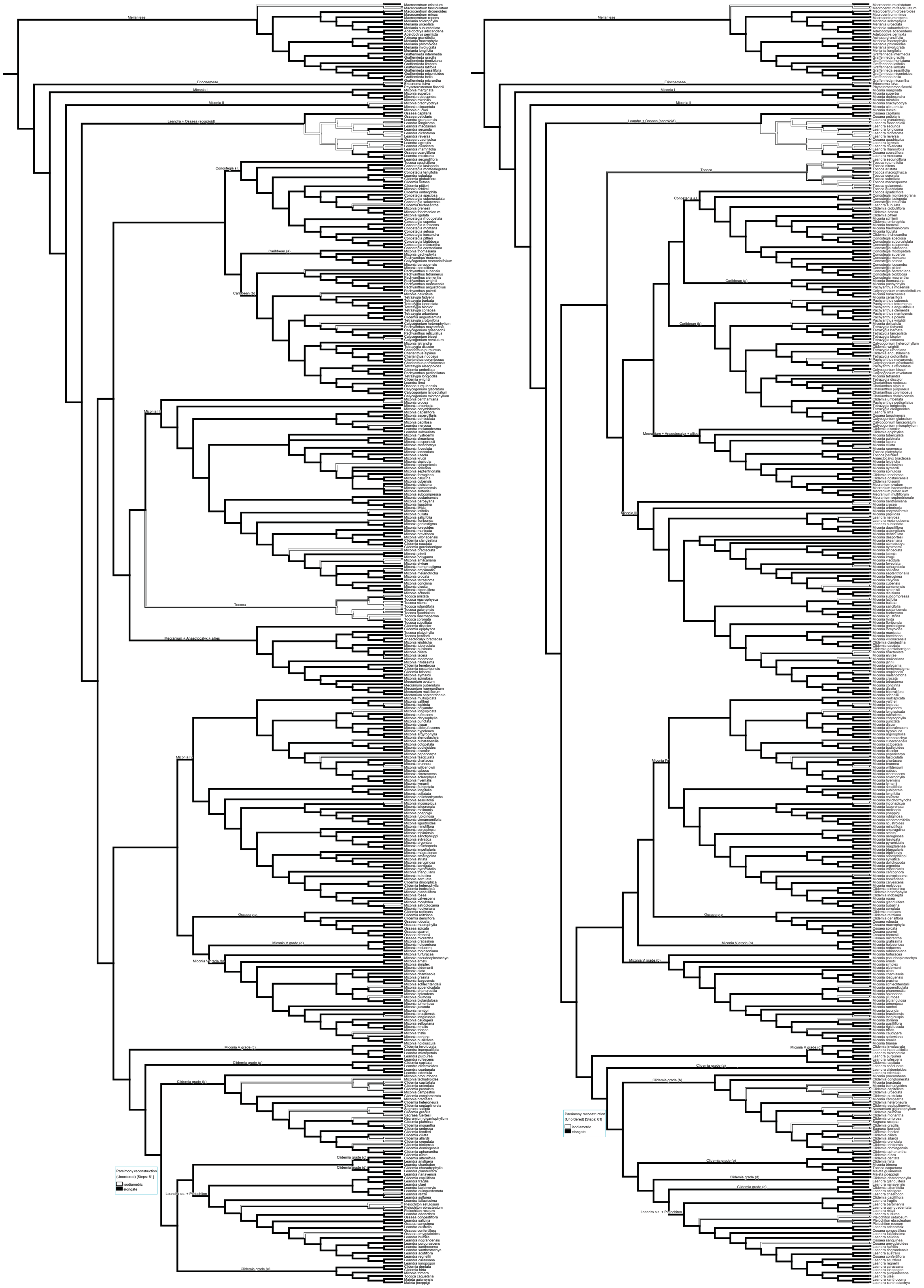

Character J. Cell shape.

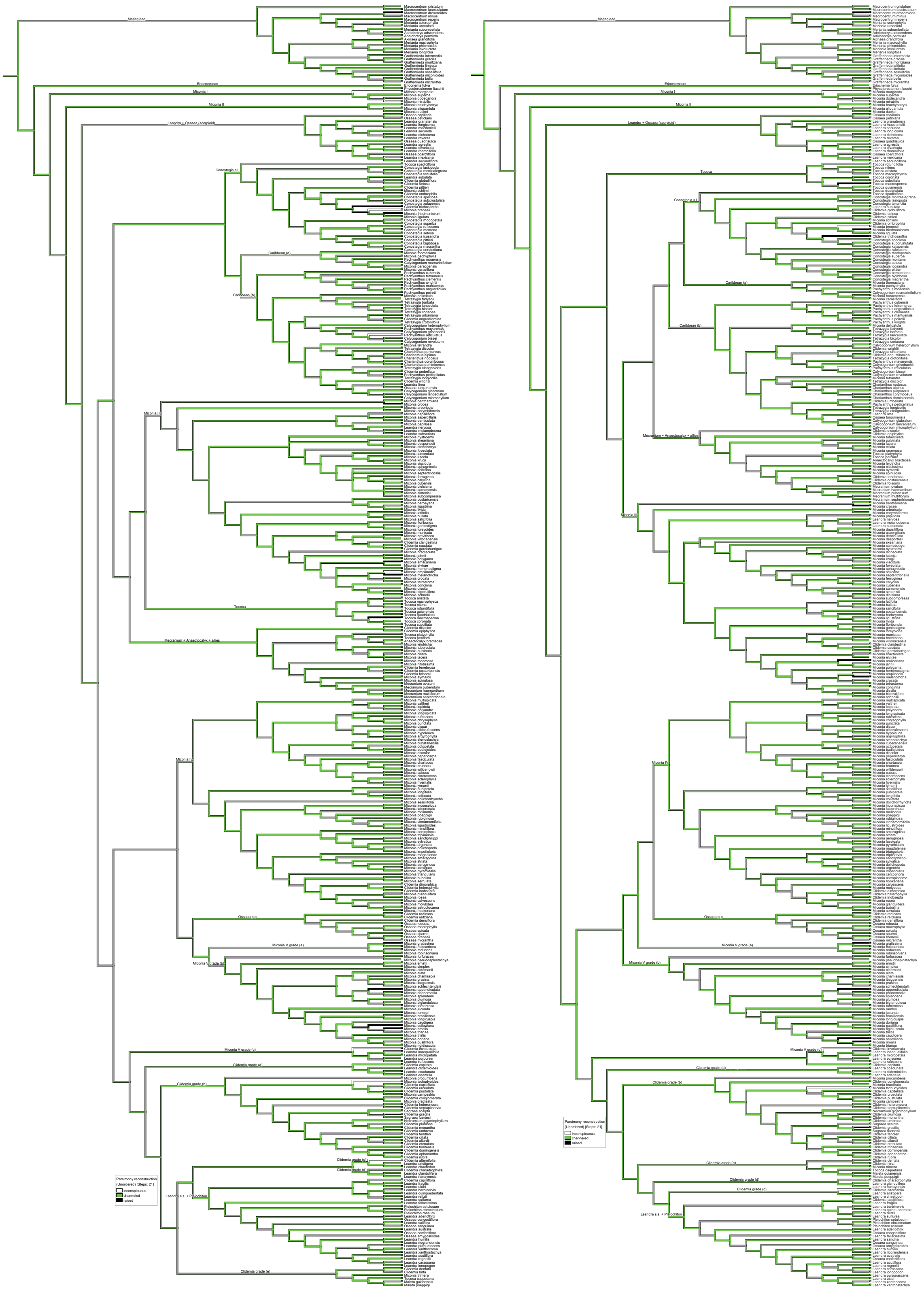

Character K. Relief of the anticlinal walls.

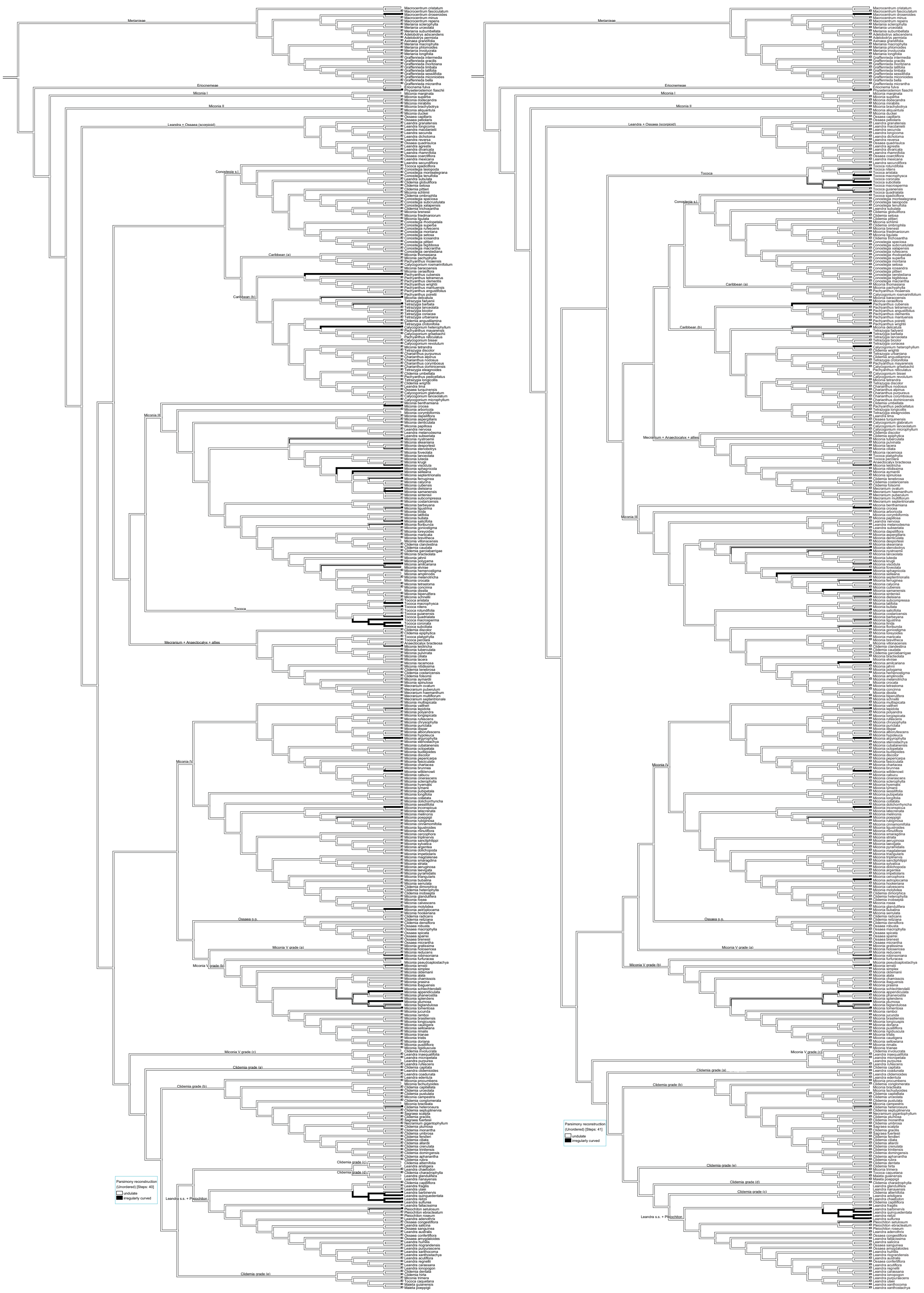

Character L. Curvature of the anticlinal walls.

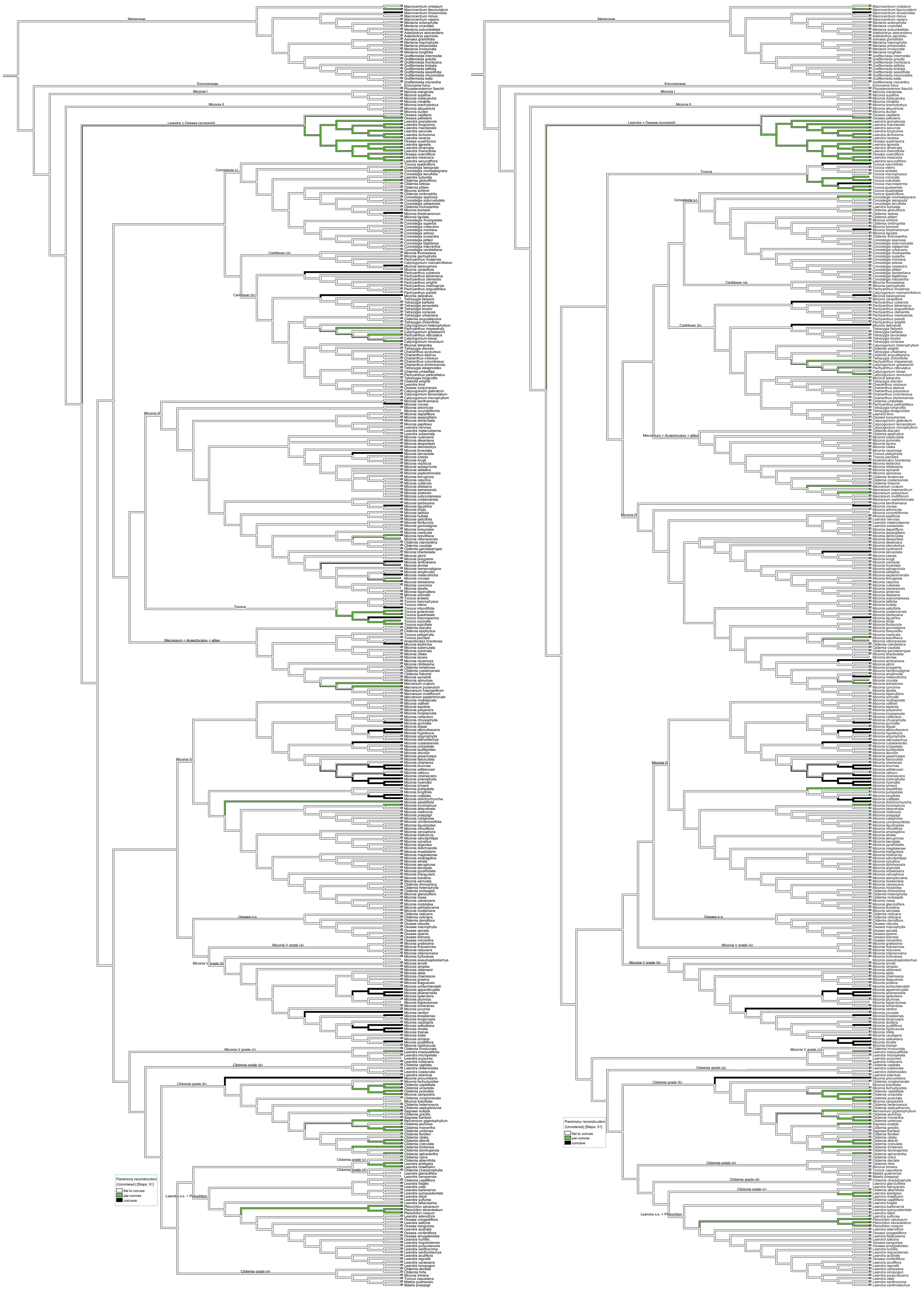

Character M. Relief of the periclinal walls.

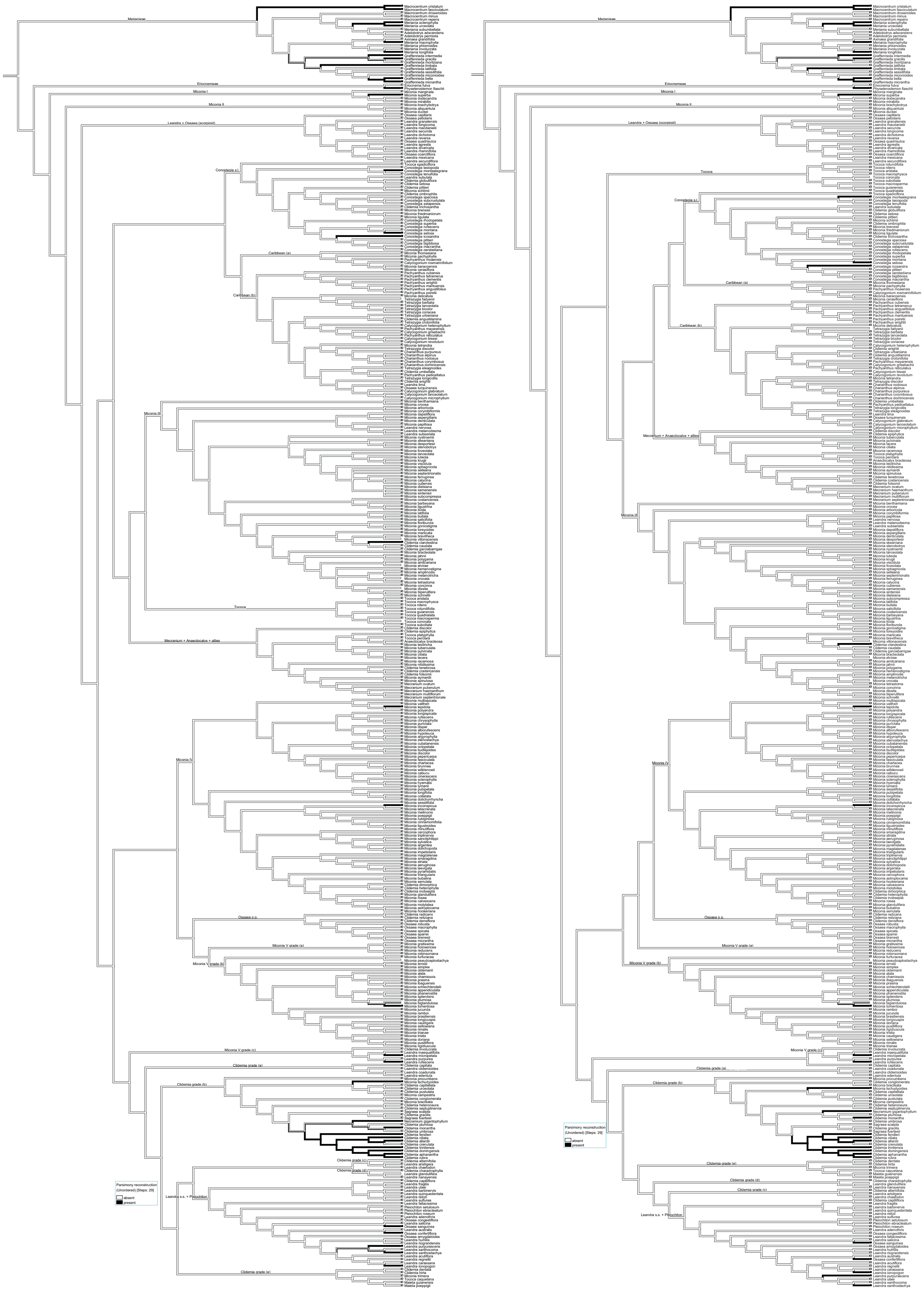

Character N. Periclinal walls dividing into two or more segments.

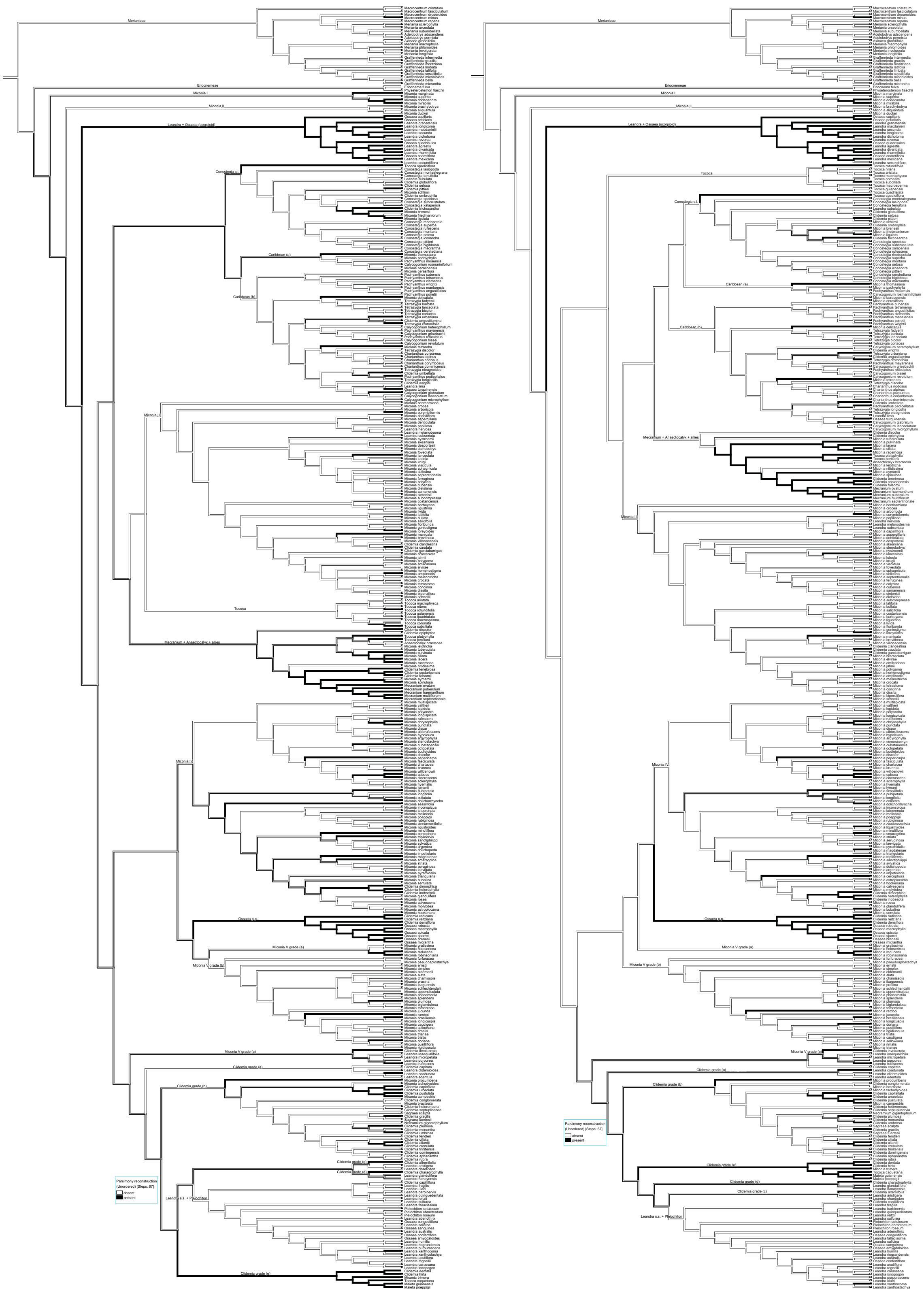

Character O. Microrelief of the periclinal walls.

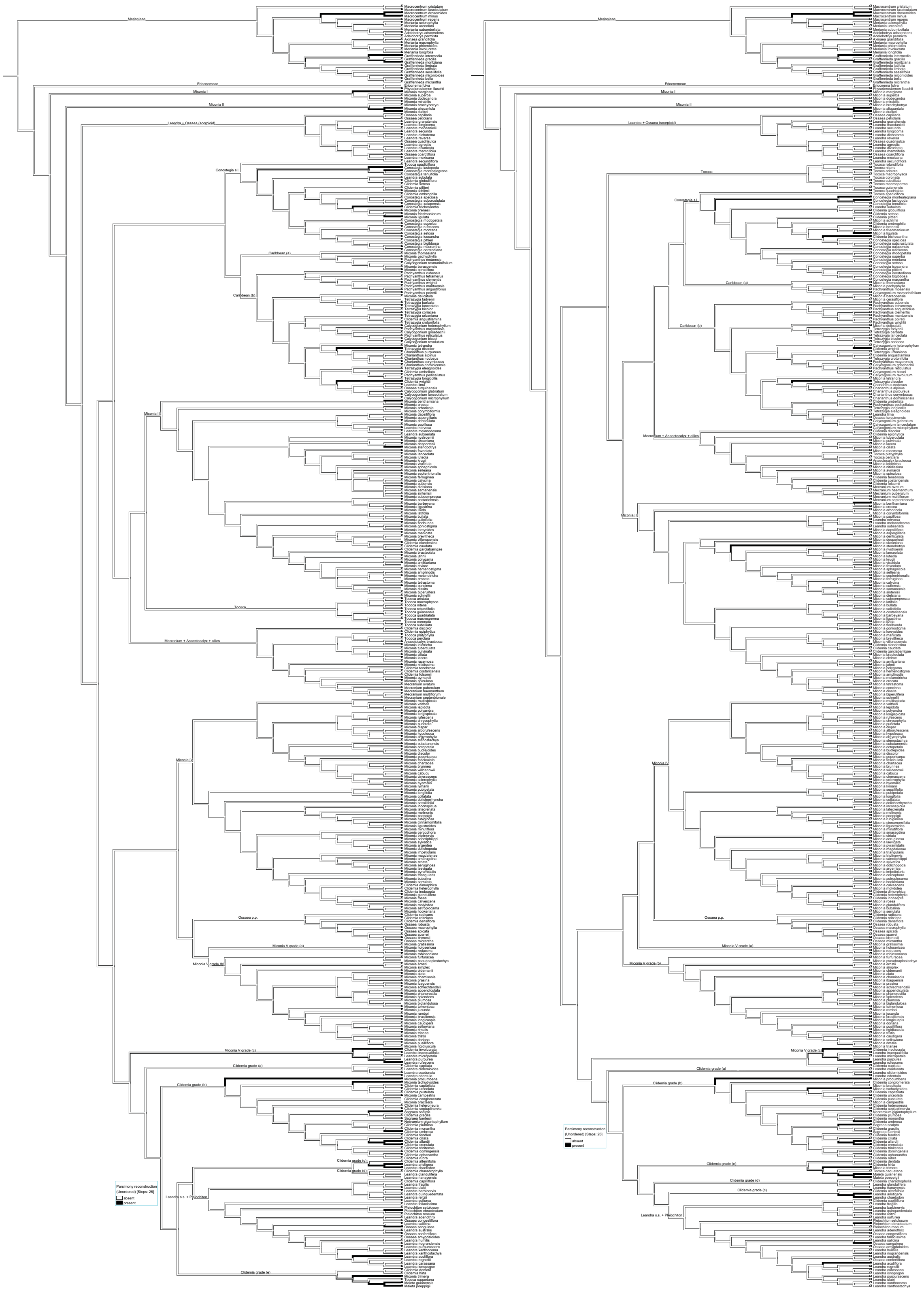

Character P. Cells with features differing from the rest of the seed corpus.

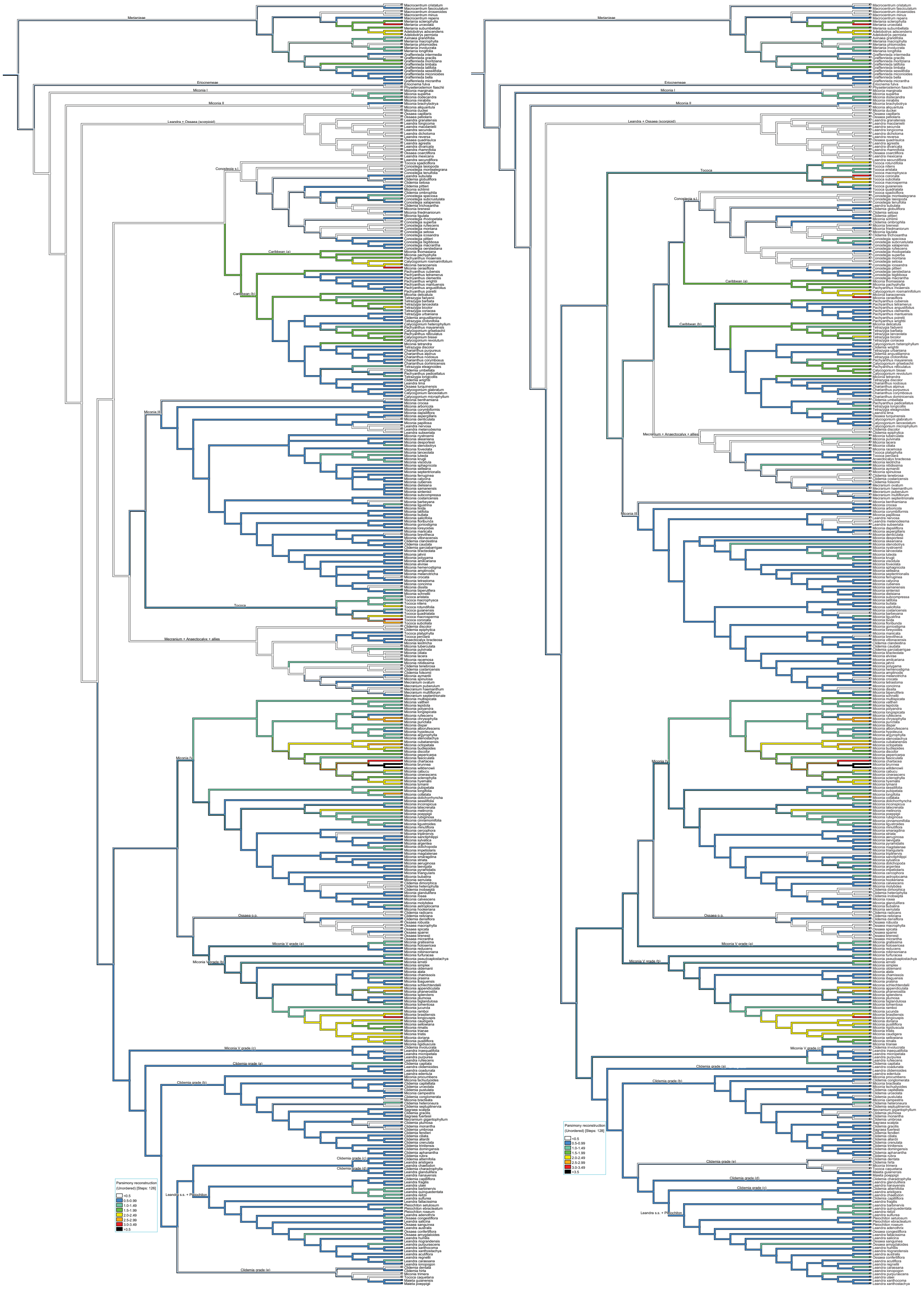

Character Q. Length of the seed from antiraphal view (mm).
