# Supplementary material for: Seed Diversity in the Tribe Miconieae (Melastomataceae): Taxonomic, Systematic, and Evolutionary Implications
Source: PLoS One. 2014 Jun 23;9(6):e100561. doi: 10.1371/journal.pone.0100561 (PMC4067357; doi:10.1371/journal.pone.0100561)
Supplement: Table S1 — Species name, source from which seed information was obtained (voucher information of plant material or literature reference), and GenBank accession numbers (ETS, ITS, accD-psaI, and psbK-psbI). Taxa are arranged in alphabetical order by genus and species. Outgroup taxa are shown at the end of the table. NA = not available; * = SEM image taken at CAS; ‡ = SEM image taken at NY. (PDF) [file pone.0100561.s003.pdf]

Table S1. Species name, source from which seed information was obtained (voucher information of plant material or literature reference), and GenBank accession numbers (ETS, ITS, *accD-psaI* , and *psbK-psbI*). Taxa are arranged in alphabetical order by genus and species. Outgroup taxa are shown at the end of the table. NA = not available; * = SEM image taken at CAS; ‡ = SEM image taken at NY.

Species	Source	ETS	ITS	*accD-psaI*	*psbK-psbI*
Anaectocalyx bracteosa (Naudin) Triana ex Cogn.	Ocampo and Almeda [31]	KF820582	AY460460	KF819864	KF821784
Calycogonium bissei Bécquer	Cuba, *HFC 11650* (HAJB)‡	KF820591	KF821408	KF819873	KF821793
Calycogonium glabratum (Sw.) DC.	Ocampo and Almeda [31]	KF820594	EU055645	KF819875	KF821795
Calycogonium grisebachii Triana	Bécquer et al. [57]	KF820596	KF821409	KF819877	KF821797
Calycogonium heterophyllum Naudin	Cuba, *HFC 81151* (HAJB)‡	KF820597	EU055647	KF819878	KF821798
Calycogonium lanceolatum Griseb.	Cuba, *HFC 84642* (HAJB)‡	KF820599	KF821410	KF819880	KF821800
Calycogonium microphyllum C.Wright	Cuba, *Ekman 12729* (NY)‡	KF820600	KF821411	KF819881	KF821801
Calycogonium revolutum Alain	Cuba, *HFC 82510* (HAJB)‡	KF820603	EU055650	KF819884	KF821804
Calycogonium rosmarinifolium Griseb.	Cuba, *León 19329* (NY)‡	KF820606	EU055651	NA	KF821807
Charianthus alpinus (Sw.) R.A.Howard	Ocampo and Almeda [31]	KF820610	AY460463	KF819891	KF821813
Charianthus corymbosus (Rich.) Cogn.	Dominica, *Cooper 31* (NY)‡	KF820611	AY460464	NA	KF821814
Charianthus dominicensis Penneys & Judd	Ocampo and Almeda [31]	KF820612	AY460465	KF819892	KF821815
Charianthus nodosus (Desr.) Triana	Ocampo and Almeda [31]	KF820613	AY460466	KF819893	KF821816
Charianthus purpureus D.Don	Ocampo and Almeda [31]	NA	AY460467	NA	NA
Clidemia allardii Wurdack	Ecuador, *Øllgaard 35365* (NY)‡	KF820615	AY460468	KF819895	KF821818
Clidemia alternifolia Wurdack	Venezuela, *Maas 6855* (NY)‡	KF820617	AY460469	NA	KF821820
Clidemia angustilamina Judd & Skean	Dominican Republic, *Judd 6662* (NY)‡	KF820619	EF418923	KF819898	KF821822
Clidemia aphanantha (Naudin) Sagot	Venezuela, *Liesner 25940* (NY)‡	KF820621	EF418797	KF819900	KF821824
Clidemia capilliflora (Naudin) Cogn.	Brazil, *Thomas 9936* (NY)‡	KF820627	EU055654	KF819905	NA
Clidemia capitata Benth.	Ocampo and Almeda [31]	KF820628	AY460471	KF819906	KF821830
Clidemia capitellata (Bonpl.) D.Don	Brazil, *Irwin 27199* (NY)‡	KF820629	EU055655	KF819907	KF821831
Clidemia caudata Wurdack	Ocampo and Almeda [31]	KF820631	KF821428	KF819908	KF821833
Clidemia charadrophylla Tutin	Ocampo and Almeda [31]	KF820632	KF821429	KF819909	KF821834
Clidemia ciliata Pav. ex D.Don	Ocampo and Almeda [31]	KF820633	AY460472	KF819910	KF821835
Clidemia clandestina Almeda	Ocampo and Almeda [31]	KF820634	EU055656	KF819911	KF821836
Clidemia conglomerata DC.	Guyana, *Clarke 828* (CAS)*	KF820635	EF418798	KF819912	KF821837
Clidemia costaricensis Cogn. & Gleason ex Gleason	Costa Rica, *Herrera 333* (NY)‡	KF820636	EU055657	KF819913	KF821838
Clidemia crenulata Gleason	Ocampo and Almeda [31]	KF820637	EF418799	NA	NA
Clidemia densiflora (Standl.) Gleason	Ocampo and Almeda [31]	KF820643	EU055658	KF819917	KF821843
Clidemia dentata D.Don	Venezuela, *Cruxent 235* (NY)‡	KF820644	EF418800	KF819918	KF821844
Clidemia dimorphica J.F.Macbr.	Peru, *McDaniel 15255* (NY)‡	KF820645	AY460476	KF819919	KF821845
Clidemia discolor (Triana) Cogn.	Ocampo and Almeda [31]	KF820646	EU055659	KF819920	KF821846
Clidemia domingensis (DC.) Cogn.	Ocampo and Almeda [31]	KF820648	EU055660	NA	KF821848
Clidemia epiphytica (Triana) Cogn.	Ecuador, *Clark 539* (NY)‡	KF820651	EF418801	KF819923	KF821851
Clidemia fendleri Cogn.	Ocampo and Almeda [31]	KF820654	AY460477	NA	KF821854
Clidemia folsomii Almeda	Panama, *Almeda 5899* (NY)‡	KF820655	EU055661	KF819926	KF821855
Clidemia garciabarrigae Wurdack	Ocampo and Almeda [31]	NA	EU055662	NA	NA
Clidemia globuliflora (Cogn.) L.O.Williams	Ocampo and Almeda [31]	KF820657	KF821437	KF819927	KF821857
Clidemia gracilis Pittier	Colombia, *Acevedo-Rodríguez 6779* (NY)‡	KF820659	KF821439	KF819929	KF821859
Clidemia heteroneura (Schrank & Mart. ex DC.) Cogn.	Brazil, *Poole 2064* (NY)‡	KF820664	AY460478	KF819932	KF821864
Clidemia heterophylla (Desr.) Gleason	NA, *Baker 5971* (NY)‡	KF820665	EU055663	KF819933	KF821865
Clidemia hirta (L.) D.Don	Brazil, *Romero 375* (NY)‡	KF820666	AY460479	KF819934	KF821866

Clidemia inobsepta Wurdack	Ocampo and Almeda [31]	KF820667	EU055664	KF819935	KF821867
Clidemia involucrata DC.	NA, *Maguire 33768* (NY)‡	KF820668	EF418804	KF819936	KF821868
Clidemia monantha L.O.Williams	Ocampo and Almeda [31]	KF820672	EU055665	NA	KF821872
Clidemia ombrophila Gleason	Costa Rica, *Almeda 5441* (NY)‡	KF820680	EU055667	KF819943	KF821880
Clidemia pittieri Gleason	Ocampo and Almeda [31]	KF820684	EU055668	KF819945	KF821884
Clidemia plumosa (Desr.) DC.	Venezuela, *Weitzman 84* (NY)‡	KF820685	EU055669	KF819946	KF821885
Clidemia pustulata DC.	Ocampo and Almeda [31]	KF820687	KF821455	KF819948	KF821887
Clidemia radicans Cogn.	Costa Rica, *Brenes 13520* (NY)‡	KF820689	EU055670	KF819950	KF821889
Clidemia reitziana Cogn. & Gleason	Ocampo and Almeda [31]	KF820690	KF821457	KF819951	KF821890
Clidemia rubra (Aublet) Martius	Ocampo and Almeda [31]	KF820692	AY460481	KF819953	KF821892
Clidemia septuplinervia Cogn.	Ecuador, *Jativa 1040* (NY)‡	KF820694	AY460482	KF819954	KF821894
Clidemia setosa (Triana) Gleason	Ocampo and Almeda [31]	KF820698	EU055671	KF819957	KF821898
Clidemia tenebrosa Almeda	Ocampo and Almeda [31]	KF820707	EU055672	KF819965	KF821907
Clidemia trichosantha Almeda	Panama, *Almeda 6491* (NY)‡	KF820708	EU055673	KF819966	KF821908
Clidemia trinitensis (Crueg.) Griseb.	Trinidad and Tobago, *Fendler 987* (NY)‡	KF820710	EF418806	NA	KF821910
Clidemia umbellata (Mill.) L.O.Williams	Ocampo and Almeda [31]	KF820711	EU055674	KF819967	KF821911
Clidemia umbrosa (Sw.) Cogn.	Ocampo and Almeda [31]	KF820712	EF418807	KF819968	KF821912
Clidemia urceolata DC.	Brazil, *Hatschbach 47432* (NY)‡	KF820713	EU055675	NA	KF821913
Clidemia wrightii Griseb.	Cuba, *HFC 79750* (HAJB)‡	KF820716	KF821470	KF819971	KF821916
Conostegia bigibbosa Cogn.	Ocampo and Almeda [31]	KF820718	AY460485	KF819973	KF821918
Conostegia icosandra (Sw. ex Wikstr.) Urb.	Guadeloupe, *Martin 488* (NY)‡	KF820719	AY460486	KF819974	KF821919
Conostegia lasiopoda Benth.	Ocampo and Almeda [31]	KF820720	KF821472	KF819975	KF821920
Conostegia macrantha O.Berg ex Triana	Ocampo and Almeda [31]	KF820721	EF418809	KF819976	KF821921
Conostegia montana (Sw.) D.Don ex DC.	Guatemala, *Stevens 25434* (NY)‡	KF820722	AY460488	KF819977	KF821922
Conostegia monteleagreana Cogn.	Panama, *Almeda 6075* (NY)‡	KF820723	EF418810	KF819978	KF821923
Conostegia oerstediana O.Berg ex Triana	Ocampo and Almeda [31]	KF820724	KF821473	KF819979	KF821924
Conostegia pittieri Cogn. ex T.Durand	Costa Rica, *Bello & Cruz 2704* (CAS)*	KF820725	EU055678	KF819980	KF821925
Conostegia rhodopetala Donn. Sm.	Costa Rica, *Schnell 1081* (NY)‡	KF820726	EU055679	KF819981	KF821926
Conostegia rufescens Naudin	Panama, *McPherson 12553* (NY)‡	KF820727	AY460489	KF819982	KF821927
Conostegia setosa Triana	Ecuador, *Freire 1060* (NY)‡	KF820728	EU055680	KF819983	KF821928
Conostegia speciosa Naudin	Ocampo and Almeda [31]	KF820729	AY460490	KF819984	KF821929
Conostegia subcrustulata (Beurling) Triana	Ocampo and Almeda [31]	KF820730	EU055681	KF819985	KF821930
Conostegia superba Naudin	Honduras, *McDougal et al. 3281* (CAS)*	KF820731	KF821474	KF819986	KF821931
Conostegia tenuifolia Donn. Sm.	Panama, *Gentry 16821* (NY)‡	KF820732	AY460491	KF819987	KF821932
Conostegia xalapensis (Bonpl.) D.Don ex DC.	Ocampo and Almeda [31]	KF820733	EU055682	KF819988	KF821933
Leandra acutiflora (Naudin) Cogn.	Brazil, *Dusen 15044* (NY)‡	KF820758	EF418813	KF820010	NA
Leandra adenothrix Cogn.	Brazil, *Irwin 26014* (NY)‡	KF820759	EF418814	NA	KF821955
Leandra agrestis (Aublet) Raddi	Ocampo and Almeda [31]	KF820760	EF418815	KF820011	KF821956
Leandra aristigera (Naudin) Cogn.	Ecuador, *Brandbyge 33882* (NY)‡	KF820762	EF418816	NA	KF821957
Leandra australis (Cham.) Cogn.	Brazil, *Sobral 1459* (NY)‡	KF820766	EU055686	KF820014	KF821960
Leandra barbinervis (Cham. ex Triana) Cogn.	Brazil, *Lacerda 271* (NY)‡	KF820767	EF418817	KF820015	KF821961
Leandra carassana (DC.) Cogn.	Brazil, *Harley 52598* (NY)‡	KF820771	EU055688	NA	KF821965
Leandra chaetodon (DC.) Cogn.	Peru, Rimachi 6980 (NY)‡	KF820776	KF821499	KF820020	KF821967
Leandra clidemioides (Naudin) Wurdack	Brazil, *Carvalho 6119* (NY)‡	KF820777	EF418820	KF820021	KF821968
Leandra coadunata Wurdack	Brazil, *Nee 42635* (NY)‡	KF820778	EF418821	KF820022	KF821969
Leandra dichotoma (Pav. ex D.Don) Cogn.	Ocampo and Almeda [31]	KF820782	EF418825	KF820026	KF821973
Leandra divaricata (Naudin) Cogn.	Ocampo and Almeda [31]	KF820783	EF418827	NA	KF821974

Leandra edentula Gleason	Guyana, *Boom 8971* (NY)‡	KF820785	EF418829	NA	KF821976
Leandra fallacissima Markgr.	Ocampo and Almeda [31]	KF820787	NA	NA	NA
Leandra fragilis Cogn.	Brazil, *Harley 20163* (NY)‡	KF820789	EF418830	KF820030	KF821980
Leandra glandulifera (Triana) Cogn.	Venezuela, *Wurdack 43448* (NY)‡	KF820791	EF418832	NA	KF821982
Leandra granatensis Gleason	Ocampo and Almeda [31]	KF820794	EU055691	KF820034	KF821985
Leandra humilis (Cogn.) Wurdack	Brazil, *Romero 10207* (NY)‡	KF820774	EF418834	KF820038	KF821988
Leandra inaequalifolia (DC) Cogn.	Brazil, *Jobert 854* (NY)‡	KF820798	EF418835	KF820039	KF821989
Leandra ionopogon (Mart.) Cogn.	Brazil, *Thomas 9280* (NY)‡	KF820799	EF418836	KF820040	KF821990
Leandra lima (Desr.) Judd & Skean	Dominican Republic, *Judd 5172* (NY)‡	KF820802	AY460493	NA	KF821994
Leandra longicoma Cogn.	Ocampo and Almeda [31]	KF820803	EF418839	NA	KF821995
Leandra macdanielii Wurdack	Ocampo and Almeda [31]	KF820807	AY460494	KF820047	KF821999
Leandra melanodesma (Naudin) Cogn.	Ocampo and Almeda [31]	KF820809	EU055695	KF820049	KF822001
Leandra mexicana (Naudin) Cogn.	Belize, *Nee et al. 46915* (CAS)*	KF820811	AY460492	NA	KF822003
Leandra micropetala (Naudin) Cogn.	Brazil, *Nee 42692* (NY)‡	KF820812	EF418842	NA	KF822004
Leandra nanayensis Wurdack	Peru, *McDaniel 20287* (NY)‡	KF820814	EF418843	KF820052	KF822006
Leandra nervosa (Naudin) Cogn.	Ecuador, *Asplund 16912* (NY)‡	NA	EU055696	KF820053	KF822007
Leandra purpurascens (DC.) Cogn.	Brazil, *Reitz 7138* (NY)‡	KF820820	EU055699	KF820058	KF822012
Leandra purpurea Gleason	Guyana, *McDowell 3588* (NY)‡	KF820821	EF418849	NA	KF822013
Leandra quinquedentata (DC.) Cogn.	Brazil, *Plowman 10136* (NY)‡	KF820823	EF418867	NA	NA
Leandra regnellii (Triana) Cogn.	Brazil, *Molon s.n.* (NY)‡	KF820826	EF418851	KF820062	KF822017
Leandra reitzii Wurdack	Brazil, *Rosa 24* (NY)‡	KF820827	EU055700	KF820063	NA
Leandra reversa (DC.) Cogn.	Ocampo and Almeda [31]	KF820828	EU055701	KF820064	KF822018
Leandra rhamnifolia (Naudin) Cogn.	Ocampo and Almeda [31]	KF820829	EU055702	KF820065	KF822019
Leandra riograndensis (Brade) Wurdack	Brazil, *Caproglione s.n.* (NY)‡	KF820830	EF418852	KF820066	KF822020
Leandra rufescens (DC.) Cogn.	Brazil, *Amorim 2378* (NY)‡	KF820833	EF418853	KF820069	KF822023
Leandra salicina (DC.) Cogn.	Brazil, *Anderson 8457* (NY)‡	KF820834	EU055703	NA	NA
Leandra secunda (D.Don) Cogn.	Brazil, *Prance 16850* (NY)‡	KF820835	AY460495	KF820070	KF822024
Leandra secundiflora (DC.) Cogn.	Brazil, *Ferreira 3600* (NY)‡	KF820836	EF418855	KF820071	KF822025
Leandra subseriata (Naudin) Cogn.	Colombia, *Fosberg 21194* (NY)‡	KF820841	AY460496	KF820075	KF822029
Leandra subulata Gleason	Ocampo and Almeda [31]	KF820842	AY460497	KF820076	KF822030
Leandra sulfurea (Naudin) Cogn.	Brazil, *Segadas-Vianna 2828* (NY)‡	KF820843	EF418863	KF820077	KF822031
Leandra ulaei Cogn.	Brazil, *Lourteig 2367* (NY)‡	KF820847	EF418865	KF820081	KF822035
Leandra xanthocoma (Naudin) Cogn.	Brazil, *Reitz 17967* (NY)‡	KF820849	EF418868	KF820083	NA
Leandra xanthostachya Cogn.	Brazil, *Anderson 36036* (NY)‡	KF820850	EF418869	KF820084	NA
Maieta guianensis Aublet	Ocampo and Almeda [31]	KF820857	AY460498	KF820093	KF822044
Maieta poeppigii Martius ex Cogn.	Ocampo and Almeda [31]	KF820858	AY460499	KF820094	KF822045
Mecranium haemanthum Triana ex Cogn.	Ocampo and Almeda [31]	KF820862	EF418871	KF820098	KF822049
Mecranium multiflorum (Desr.) Triana	Haiti, *Skean 1631* (NY)*	KF820867	EF418872	KF820103	KF822053
Mecranium ovatum Cogn.	Ocampo and Almeda [31]	KF820869	EU055706	KF820105	KF822055
Mecranium puberulum Cogn.	Dominican Republic, *Zanoni 28316* (NY)‡	KF820870	EF418873	KF820106	KF822056
Mecranium septentrionale Skean	Ocampo and Almeda [31]	KF820872	KF821535	KF820108	KF822058
Miconia aeruginosa Naudin	Ocampo and Almeda [31]	KF820895	AY460501	KF820128	KF822081
Miconia alata (Aublet) DC.	Ocampo and Almeda [31]	KF820908	KF821553	KF820135	KF822090
Miconia alborufescens Naudin	Brazil, *Prance P24922* (NY)‡	KF820910	EU055714	KF820138	KF822093
Miconia aliquantula Wurdack	Ocampo and Almeda [31]	KF820911	EF418881	KF820139	KF822094
Miconia amilcariana Almeda & Dorr	Venezuela, *Dorr et al. 8416* (CAS)*	KF820971	EU055746	KF820195	KF822156
Miconia amplinodis Umaña & Almeda	Ocampo and Almeda [31]	KF820915	EU055715	KF820143	KF822098

Miconia appendiculata Triana	Costa Rica, *Davidse & Herrera 26232* (CAS)*	KF820920	EU055716	KF820148	KF822103
Miconia arboricola Almeda	Ocampo and Almeda [31]	KF820922	EU055717	KF820150	KF822105
Miconia argentea (Sw.) DC.	Ocampo and Almeda [31]	KF820923	AY460503	KF820151	KF822106
Miconia argyrophylla DC.	Ocampo and Almeda [31]	KF820925	EF418882	KF820153	KF822108
Miconia aspergillaris (Bonpl.) Naudin	Ecuador, *Boeke 2475* (NY)‡	KF820896	EU055709	KF820129	KF822082
Miconia astroplocama Donnell Smith	Ocampo and Almeda [31]	KF820927	EU055719	KF820155	KF822110
Miconia aymardii Wurdack	Ocampo and Almeda [31]	KF820972	NA	KF820276	KF822246
Miconia baracoensis Urb.	Bécquer et al. [57]	KF820934	EF418883	KF820161	KF822117
Miconia barbeyana Cogn.	Peru, *Smith 5593* (NY)‡	KF820935	EU055721	KF820162	KF822118
Miconia benthamiana Triana	Ocampo and Almeda [31]	KF820937	EU055722	KF820164	KF822120
Miconia biglandulosa Gleason	Peru, *Bernardi 9/11* (NY)‡	KF820939	EU055723	KF820166	KF822122
Miconia biperulifera Cogn.	Ocampo and Almeda [31]	NA	EU055724	NA	KF822124
Miconia brachybotrya Triana	Ocampo and Almeda [31]	KF820942	EU055725	KF820168	KF822126
Miconia bracteata (DC.) Triana	Brazil, *Mori 17199* (NY)‡	KF820943	EF418884	KF820169	KF822127
Miconia bracteolata (Bonpl.) DC.	Ocampo and Almeda [31]	KF820944	EU055726	KF820170	KF822128
Miconia brasiliensis (Spreng.) Triana	Brazil, *Klein 107* (NY)‡	KF820945	EU055727	NA	NA
Miconia brenesii Standl.	Costa Rica, *Penneys 269* (CAS)*	KF820946	EU055728	KF820171	KF822129
Miconia brevitheca Gleason	Ocampo and Almeda [31]	KF820947	EU055729	KF820172	KF822130
Miconia brunnea DC.	Ocampo and Almeda [31]	KF820948	EU055730	KF820173	KF822131
Miconia bubalina (D.Don) Naudin	Ocampo and Almeda [31]	KF820949	EU055731	NA	KF822132
Miconia buddlejoides Triana	Brazil, *Klein 1239* (NY)‡	KF820950	EU055732	KF820174	KF822133
Miconia bullata (Turcz.) Triana	Ocampo and Almeda [31]	KF820951	EU055733	KF820175	KF822134
Miconia cabucu Hoehne	Brazil, *Dusen 17341* (NY)‡	KF820952	EU055734	NA	KF822135
Miconia calvescens DC.	Ocampo and Almeda [31]	KF820955	NA	KF820178	KF822138
Miconia calycina Cogn.	Ocampo and Almeda [31]	KF820956	EU055737	KF820179	KF822139
Miconia campestris (Benth.) Triana	Ocampo and Almeda [31]	KF820957	KF821573	NA	KF822140
Miconia caudigera DC.	Ocampo and Almeda [31]	KF820963	EU055740	KF820185	KF822146
Miconia cerasiflora Urb.	Cuba, *HFC 82487* (HAJB)‡	KF820968	EU055743	KF820191	KF822152
Miconia cercophora Wurdack	Ocampo and Almeda [31]	KF820969	EU055745	KF820192	KF822153
Miconia chamissois Naudin	El Salvador, *Monterrosa & Carballo 791* (CAS)*	KF820974	EU055748	KF820197	KF822158
Miconia chartacea Triana	Ocampo and Almeda [31]	KF820975	EU055749	KF820198	KF822159
Miconia chrysophylla (Richard) Urban	Ocampo and Almeda [31]	KF820977	EU055750	KF820200	KF822161
Miconia ciliata (Rich.) DC.	Venezuela, *Tate 1133* (NY)‡	KF820979	KF821581	KF820201	KF822162
Miconia cinerascens Miquel	Ocampo and Almeda [31]	KF820981	EU055751	KF820202	KF822164
Miconia cinnamomifolia (DC.) Naudin	Brazil, *Martins 6680* (NY)‡	KF820982	EU055753	KF820204	KF822166
Miconia collatata Wurdack	Brazil, *Prance 59642* (NY)‡	KF820986	EU055754	KF820207	KF822170
Miconia concinna Almeda	Panama, *McPherson 11373* (CAS)*	KF820988	EU055755	KF820209	KF822172
Miconia corymbiformis Cogn.	Ecuador, *Ortiz et al. 307* (CAS)*	KF820992	EU055756	KF820213	KF822176
Miconia costaricensis Cogn.	Ocampo and Almeda [31]	KF820993	EU055757	KF820214	KF822177
Miconia crocata Almeda	Panama, *Almeda et al. 6388* (CAS)*	KF820997	EU055759	KF820217	KF822181
Miconia crocea (Desr.) Naudin	Ecuador, *Harling & Andersson 23467* (CAS)*	KF820998	EU055760	KF820218	KF822182
Miconia cubatanensis Hoehne	Brazil, *Mexia 5776* (NY)‡	KF820999	EU055761	KF820219	KF822183
Miconia cubensis (C.Wright ex Griseb.) C.Wright	Ocampo and Almeda [31]	KF821000	EU055762	KF820220	KF822184
Miconia dapsiliflora Wurdack	Ecuador, *Penneys 1898* (FLAS)‡	KF821006	EU055763	KF820225	KF822189
Miconia delicatula A.Rich.	Cuba, *HFC 82421* (HAJB)‡	NA	EU055764	NA	NA
Miconia denticulata Naudin	Ocampo and Almeda [31]	KF821009	EU055765	KF820228	KF822192
Miconia desportesii Urban	Ocampo and Almeda [31]	KF821011	EF418887	KF820229	KF822194

Miconia dielsiana Urban	Ocampo and Almeda [31]	KF821014	EU055766	KF820232	KF822197
Miconia discolor DC.	Argentina, *Mulgura 1918* (NY)‡	KF821015	EU055767	KF820233	KF822198
Miconia dispar Benth.	Ocampo and Almeda [31]	KF821016	KF821597	KF820234	KF822199
Miconia dissita Almeda	Panama, *Croat & Zhu 76484* (CAS)*	KF821017	EU055768	KF820235	KF822200
Miconia dodecandra Cogn.	Ocampo and Almeda [31]	KF821020	KF821600	NA	NA
Miconia dolichopoda Naudin	Ocampo and Almeda [31]	KF821022	KF821601	KF820239	KF822204
Miconia dolichorrhyncha Naudin	Ocampo and Almeda [31]	KF821023	KF821602	KF820240	KF822205
Miconia doriana Cogn.	Brazil, *Fiaschi 1375* (NY)‡	KF821025	EU055771	KF820241	KF822207
Miconia duckei Cogn.	Ocampo and Almeda [31]	KF821028	AY460508	NA	KF822210
Miconia elvirae Wurdack	Venezuela, *Dorr et al. 5012* (NY)*	KF821033	EU055773	KF820248	KF822215
Miconia ernstii Wurdack	Ocampo and Almeda [31]	KF821036	EF418889	KF820251	KF822218
Miconia fasciculata Gardner	Brazil, *Kozera 1427* (NY)‡	KF821037	EU055774	KF820252	KF822219
Miconia ferruginea (Desr.) DC.	Ocampo and Almeda [31]	KF821039	KF821611	KF820253	KF822221
Miconia floribunda (Bonpl.) DC.	NA, *Rusby 792* (NY)‡	KF821040	EU055775	KF820254	KF822222
Miconia foveolata Cogn.	Ocampo and Almeda [31]	KF821041	AY460511	KF820255	KF822223
Miconia friedmaniorum Almeda & Umaña	Ocampo and Almeda [31]	KF821042	EU055776	KF820257	KF822225
Miconia furfuracea (Vahl) Griseb.	Ocampo and Almeda [31]	KF821044	EU055777	KF820260	KF822228
Miconia glandulifera Cogn.	Ocampo and Almeda [31]	KF821046	KF821614	KF820262	KF822230
Miconia goniostigma Triana	Panama, *Sytsma 1513* (CAS)*	KF821050	EU055778	KF820266	KF822234
Miconia gratissima Benth. ex Triana	Ocampo and Almeda [31]	KF821054	KF821620	KF820269	KF822238
Miconia hemenostigma Naudin	Ocampo and Almeda [31]	KF820900	EU055780	KF820271	KF822240
Miconia holosericea (L.) DC.	Ocampo and Almeda [31]	KF821057	KF821623	NA	KF822242
Miconia hookeriana Triana	Ocampo and Almeda [31]	KF821059	EU055781	KF820274	KF822244
Miconia hyemalis St.-Hil. & Naudin	Ocampo and Almeda [31]	KF820973	EU055782	KF820194	KF822155
Miconia hypoleuca (Benth.) Triana	Ocampo and Almeda [31]	KF821062	EU055784	KF820278	KF822248
Miconia ibaguensis (Bonpl.) Triana	Ocampo and Almeda [31]	KF821063	EU055785	KF820279	KF822249
Miconia impetiolaris (Sw.) D.Don ex DC.	Dominican Republic, *Zanoni et al. 41170* (CAS)*	KF821064	AY460513	KF820280	KF822250
Miconia inconspicua Miquel	Ocampo and Almeda [31]	KF821065	EU055786	KF820281	KF822251
Miconia jahnii Pittier	Ocampo and Almeda [31]	KF821069	KF821628	NA	KF822255
Miconia jucunda (DC.) Triana	Ocampo and Almeda [31]	KF821073	EU055789	KF820288	KF822259
Miconia krugii Cogn.	Ocampo and Almeda [31]	KF821077	EF418892	KF820290	KF822263
Miconia lacera (Bonpl.) Naudin	Ocampo and Almeda [31]	KF821079	AY460514	NA	KF822265
Miconia laevigata (L.) DC.	Ocampo and Almeda [31]	NA	AY460515	KF820292	KF822266
Miconia lanceolata (Desr.) DC.	Ocampo and Almeda [31]	KF821080	KF821634	KF820293	KF822267
Miconia latecrenata (DC.) Naudin	Ocampo and Almeda [31]	KF821082	KF821636	KF820296	KF822270
Miconia latifolia (D.Don) Naudin	Ocampo and Almeda [31]	KF821084	EF208214	KF820297	KF822271
Miconia leiotricha Wurdack	Ocampo and Almeda [31]	KF821085	AY46046	KF820298	KF822272
Miconia lepidota DC.	Ocampo and Almeda [31]	KF821088	EU055792	KF820300	KF822275
Miconia ligulata Almeda	Ocampo and Almeda [31]	KF821090	EF418894	KF820302	KF822277
Miconia ligustrina (Smith) Triana	Ocampo and Almeda [31]	KF821091	EU055793	KF820303	KF822278
Miconia ligustroides (DC.) Naudin	Brazil, *Smith 12332* (NY)‡	KF821092	EU055794	KF820304	KF822279
Miconia livida Triana	Ocampo and Almeda [31]	KF821093	KF821639	KF820305	KF822280
Miconia longicuspis Cogn.	Ocampo and Almeda [31]	KF821097	EU055796	KF820309	KF822284
Miconia longifolia (Aubl.) DC.	Guyana, *Clarke 2733* (NY)‡	KF821098	EF418895	KF820310	KF822285
Miconia longispicata Triana	Ocampo and Almeda [31]	KF821099	EU055797	KF820311	KF822286
Miconia loreyoides Triana	Ocampo and Almeda [31]	KF821100	EU055798	KF820312	KF822287
Miconia luteola Cogn.	Ocampo and Almeda [31]	KF821103	EU055799	KF820315	KF822290

Miconia lymanii Wurdack	Ocampo and Almeda [31]	KF821105	EU055800	KF820317	KF822292
Miconia magdalenae Triana	Ocampo and Almeda [31]	KF821108	EF418896	KF820320	KF822295
Miconia manicata Cogn. & Gleason	Ocampo and Almeda [31]	KF821109	EU055801	KF820321	KF822296
Miconia marginata Triana	Ocampo and Almeda [31]	KF821110	KF821650	KF820325	KF822300
Miconia melanotricha (Triana) Gleason	Ocampo and Almeda [31]	KF821114	EU055802	KF820326	KF822301
Miconia melinonis Naudin	Brazil, *Santos 238* (NY)*	KF821115	EF418898	KF820327	KF822302
Miconia minutiflora (Bonpl.) DC.	Brazil, *Maguire 40254* (NY)‡	KF821124	KF821656	KF820335	KF822310
Miconia mirabilis (Aubl.) L.O.Williams	Dominica, *Wilbur et al. 8190* (CAS)*	KF821125	EU055806	KF820337	KF822312
Miconia molybdea Naudin	Ocampo and Almeda [31]	KF821127	KF821658	KF820339	KF822314
Miconia multispicata Naudin	Ocampo and Almeda [31]	KF821130	EU055808	KF820342	KF822317
Miconia nitidissima Cogn.	Ocampo and Almeda [31]	KF821134	EU055809	NA	KF822322
Miconia nystroemii Ekman ex Urban	Ocampo and Almeda [31]	KF821137	KF821666	KF820349	KF822325
Miconia octopetala Cogn.	Ocampo and Almeda [31]	KF821140	EU055810	KF820352	KF822328
Miconia oldemanii Wurdack	Ocampo and Almeda [31]	KF821142	EF418899	KF820353	KF822330
Miconia pachyphylla Cogn.	Ocampo and Almeda [31]	KF821145	AY460519	KF820356	KF822333
Miconia papillosa (Desr.) Naudin	Ocampo and Almeda [31]	KF821147	EU055812	KF820358	KF822335
Miconia pepericarpa Mart. ex DC.	Brazil, *Irwin 27393* (NY)‡	NA	EU055814	NA	NA
Miconia phanerostila Pilger	Ocampo and Almeda [31]	KF821155	KF821677	KF820366	KF822343
Miconia plumosa Gleason	Guyana, *Forest Department F1321* (NY)*	KF821160	EU055817	KF820371	KF822347
Miconia poeppigii Triana	Ocampo and Almeda [31]	KF821161	EU055818	KF820372	KF822348
Miconia polyandra Gardner	Ocampo and Almeda [31]	KF821163	EU055819	KF820374	KF822350
Miconia polygama Cogn.	Ocampo and Almeda [31]	KF821164	KF821682	KF820375	KF822351
Miconia prasina (Sw.) DC.	Ocampo and Almeda [31]	KF821165	AY460520	KF820376	KF822352
Miconia procumbens (Gleason) Wurdack	Ocampo and Almeda [31]	KF821167	AY460521	KF820377	KF822354
Miconia pseudoaplostachya Cogn.	Guyana, *Clarke et al. 2120* (CAS)*	KF821168	EF418900	KF820378	KF822355
Miconia pubipetala Miquel	Ocampo and Almeda [31]	KF821172	EF418901	KF820380	KF822359
Miconia pulvinata Gleason	Ocampo and Almeda [31]	KF821174	AY460522	KF820381	KF822360
Miconia punctata (Desr.) D.Don ex DC.	Ocampo and Almeda [31]	KF821175	EU055821	KF820382	KF822361
Miconia pusilliflora (DC.) Naudin	Ocampo and Almeda [31]	KF821176	EU055822	KF820384	KF822363
Miconia pyramidalis (Desr.) DC.	Ocampo and Almeda [31]	KF821178	EF418902	KF820386	KF822365
Miconia racemosa (Aubl.) DC.	Guyana, *Redden 1086* (NY)‡	KF821182	EU055823	KF820389	KF822369
Miconia ramboi Brade	Ocampo and Almeda [31]	KF821185	EU055824	KF820391	KF822371
Miconia reducens Triana	Ocampo and Almeda [31]	KF821186	EU055825	NA	KF822372
Miconia rigidiuscula Cogn.	Brazil, *Fritsch et al. 1810* (CAS)*	KF821190	EU055826	KF820395	KF822376
Miconia rimalis Naudin	Ocampo and Almeda [31]	KF821191	EU055827	KF820396	KF822377
Miconia robinsoniana Cogn.	Ecuador, *Stewart 3044* (CAS)*	KF821192	EU055828	KF820397	KF822378
Miconia rosea Gleason	Ocampo and Almeda [31]	KF821194	KF821695	KF820398	KF822380
Miconia rubiginosa (Bonpl.) DC.	Ocampo and Almeda [31]	KF821196	AY460525	KF820399	KF822382
Miconia rufescens (Aublet) DC.	Ocampo and Almeda [31]	KF821198	AY460526	KF820401	KF822384
Miconia salicifolia (Bonpl. ex Naudin) Naudin	Ecuador, *Holm-Nielsen et al. 29021* (CAS)*	NA	EU055831	NA	KF822388
Miconia samanensis Urban	Ocampo and Almeda [31]	KF821202	EU055832	KF820404	KF822389
Miconia sanctiphilippi Naudin	Ocampo and Almeda [31]	KF821203	AY46052	KF820405	KF822390
Miconia schlechtendalii Cogn.	Mexico, *Breedlove 37751* (CAS)*	KF821204	EU055833	KF820406	KF822391
Miconia schlimii Triana	Ocampo and Almeda [31]	KF821205	EU055834	KF820407	KF822392
Miconia schnellii Wurdack	Ocampo and Almeda [31]	KF821206	AY460528	KF820408	KF822393
Miconia sclerophylla Triana	Ocampo and Almeda [31]	KF821208	EU055835	KF820409	KF822395
Miconia selleana Urban & Ekman	Ocampo and Almeda [31]	KF821209	EF418904	KF820410	KF822396

Miconia sellowiana Naudin	Ocampo and Almeda [31]	KF821210	EU055836	KF820411	KF822397
Miconia septentrionalis Judd & R.S.Beaman	Ocampo and Almeda [31]	KF821211	EU055837	KF820412	KF822398
Miconia serrulata (DC.) Naudin	Ocampo and Almeda [31]	KF821212	AY460535	KF820413	KF822399
Miconia sessilifolia Naudin	Ocampo and Almeda [31]	KF821213	KF821700	KF820414	KF822400
Miconia simplex Triana	Panama, *McPherson 20147* (CAS)*	KF821214	EU055838	KF820415	KF822401
Miconia sintenisii Cogn.	Ocampo and Almeda [31]	KF821215	AY460529	KF820416	KF822402
Miconia skeaniana Judd	Ocampo and Almeda [31]	KF821216	EU055839	KF820417	KF822403
Miconia smaragdina Naudin	Ocampo and Almeda [31]	KF821217	EU055840	NA	KF822404
Miconia sphagnicola Urban & Ekman	Ocampo and Almeda [31]	KF821221	EU055841	KF820418	KF822405
Miconia spinulosa Naudin	Venezuela, *Dorr 7739* (NY)‡	KF821222	AY460530	KF820419	KF822406
Miconia splendens (Sw.) Griseb.	Ocampo and Almeda [31]	KF821223	KF821701	KF820420	KF822407
Miconia stenobotrys (Richard) Naudin	Ocampo and Almeda [31]	KF821227	EU055842	KF820426	KF822414
Miconia stenostachya DC.	Ocampo and Almeda [31]	KF821229	EU055843	KF820428	KF822416
Miconia striata (Vahl) Cogn.	Dominica, *Barrier 3677* (NY)*	KF821231	EU055844	KF820430	KF822418
Miconia subcompressa Urban	Ocampo and Almeda [31]	KF821233	EU055845	KF820432	KF822420
Miconia superba Ule	Ocampo and Almeda [31]	KF821234	EU055846	KF820433	KF822421
Miconia sylvatica (Schltdl.) Naudin	Ocampo and Almeda [31]	KF821235	EU055847	KF820434	KF822422
Miconia tetrandra (Sw.) D.Don	Ocampo and Almeda [31]	KF821239	EU055848	KF820438	KF822426
Miconia tetrastoma Naudin	Ocampo and Almeda [31]	KF821240	AY460532	KF820439	KF822427
Miconia thomasiana DC.	Ocampo and Almeda [31]	KF821244	EU055850	KF820443	KF822431
Miconia tomentosa (Richard) D.Don ex DC.	Ocampo and Almeda [31]	KF821248	EF418905	NA	KF822435
Miconia trianae Cogn.	Ocampo and Almeda [31]	KF821253	EU055851	KF820450	KF822440
Miconia triangularis Gleason	Ocampo and Almeda [31]	KF821254	EU055852	KF820451	KF822441
Miconia trimera Wurdack	Ocampo and Almeda [31]	KF821255	EF418907	KF820452	KF822442
Miconia triplinervis Ruiz & Pav.	Ocampo and Almeda [31]	KF821257	EU055854	KF820454	KF822444
Miconia tristis Spring	Brazil, *Brade 16594* (NY)‡	KF821258	EU055855	KF820455	KF822445
Miconia tschudyoides Cogn.	French Guiana, *Loubry 1345* (NY)‡	KF821259	EF418908	KF820456	KF822446
Miconia tuberculata (Naudin) Triana	Ocampo and Almeda [31]	KF821260	AY460534	KF820457	KF822447
Miconia valtheri Naudin	Ocampo and Almeda [31]	KF821267	EU055857	KF820464	KF822454
Miconia villonacensis Wurdack	Ecuador, *Camp E-226* (NY)*	KF820902	EU055712	KF820131	KF822084
Miconia viscidula Urban & Cogn.	Ocampo and Almeda [31]	KF821269	EF418910	KF820466	KF822456
Miconia willdenowii Klotzsch ex Naudin	Ocampo and Almeda [31]	KF821272	EU055858	KF820469	KF822459
Necramium gigantophyllum Britton	Ocampo and Almeda [31]	KF821274	AY460537	KF820471	KF822461
Ossaea amygdaloides (DC.) Triana	Brazil, *Gibbs 3525* (NY)‡	KF821277	KF821731	KF820474	KF822464
Ossaea brenesii Standl.	Ocampo and Almeda [31]	KF821284	EU055860	KF820480	KF822470
Ossaea capillaris (D.Don) Cogn.	Ocampo and Almeda [31]	KF821285	EU055861	KF820481	KF822471
Ossaea coarctiflora Wurdack	NA, *Prance 1470* (NY)‡	KF821286	EF418911	KF820482	KF822472
Ossaea confertiflora (DC.) Triana	Brazil, *Goldenberg 766* (NY)‡	KF821287	KF821737	KF820483	KF822473
Ossaea congestiflora Cogn.	Brazil, *Irwin 12247* (NY)‡	KF821288	EF418912	KF820484	KF822474
Ossaea macrophylla (Benth.) Cogn.	Ocampo and Almeda [31]	KF821296	EU055863	KF820490	NA
Ossaea micrantha (Sw.) Macfad. ex Cogn.	Ocampo and Almeda [31]	KF821299	AY460539	KF820493	KF822484
Ossaea petiolaris (Naudin) Triana	Bolivia, *Solomon 7529* (NY)‡	KF821306	EF418914	KF820500	KF822491
Ossaea quadrisulca (Naudin) Wurdack	Ocampo and Almeda [31]	KF821308	EF418915	KF820502	KF822493
Ossaea robusta (Triana) Cogn.	Ocampo and Almeda [31]	KF821309	AY460538	KF820503	KF822494
Ossaea sanguinea Cogn.	Brazil, *Pirani 763* (NY)‡	KF821313	EU055865	KF820505	NA
Ossaea sparrei Wurdack	Ecuador, *Clemants 1694* (NY)‡	KF821314	EU055867	KF820506	KF822498
Ossaea spicata Gleason	Ocampo and Almeda [31]	KF821315	EU055868	KF820507	KF822499

Ossaea turquinensis Urb.	Cuba, *Chrysogone 5814* (NY)‡	KF821317	NA	KF820508	KF822500
Pachyanthus angustifolius Griseb.	Cuba, *HFC 81592* (HAJB)‡	KF821321	EU055870	KF820510	KF822504
Pachyanthus clementis P.Wilson	Cuba, *HFC 82465* (HAJB)‡	KF821323	EU055872	KF820512	KF822506
Pachyanthus cubensis A.Rich.	Cuba, *HFC 82418* (HAJB)‡	KF821324	EU055873	KF820513	KF822507
Pachyanthus mantuensis Britton & P.Wilson	Cuba, *HFC 82419* (HAJB)‡	KF821327	EU055876	KF820516	KF822510
Pachyanthus mayarensis Urb.	Cuba, *HFC 82496* (HAJB)‡	KF821328	EU055877	KF820517	KF822511
Pachyanthus moaensis Borhidi	Cuba, *HFC 82504* (HAJB)‡	KF821329	EF418918	KF820518	KF822512
Pachyanthus pedicellatus Urb.	Cuba, *HFC 81126* (HAJB)‡	KF821332	EU055881	KF820521	KF822515
Pachyanthus poiretii Griseb.	Cuba, *HFC 81675* (HAJB)‡	KF821333	EU055882	KF820522	KF822516
Pachyanthus reticulatus Britton & P.Wilson	Cuba, *HFC 82484* (HAJB)‡	KF821334	EU055883	KF820523	KF822517
Pachyanthus tetramerus Urb. & Ekman	Cuba, *HFC 82425* (HAJB)‡	KF821335	EU055884	KF820524	KF822518
Pachyanthus wrightii Griseb.	Cuba, *HFC 82269* (HAJB)‡	KF821336	EU055885	KF820525	KF822519
Pleiochiton ebracteatum Triana	Brazil, *Reginato 1238* (NY)‡	KF821345	EF418919	NA	NA
Pleiochiton roseum Cogn.	Brazil, *Chiavegatto 143* (RB)‡	KF821348	NA	NA	NA
Pleiochiton setulosum Cogn.	Brazil, *Reginato 1122* (NY)‡	KF821349	NA	NA	NA
Sagraea fuertesii (Cogn.) Alain	Ocampo and Almeda [31]	KF820656	AY460541	NA	KF821856
Sagraea scalpta (Vent.) Naudin	Ocampo and Almeda [31]	KF821353	KF821763	KF820534	KF822526
Tetrazygia barbata Borhidi	Cuba, *HFC 85468* (HAJB)‡	KF821357	NA	KF820538	KF822530
Tetrazygia bicolor Cogn.	Cuba, *HFC 82286* (HAJB)‡	KF821358	AY460543	KF820539	KF822531
Tetrazygia coriacea Urb.	Cuba, *Liogier 1199* (NY)‡	KF821359	EU055891	KF820540	KF822532
Tetrazygia crotonifolia (Desr.) DC.	Cuba, *Williams & Whitten s.n.* (FLAS)‡	KF821361	KF821766	KF820542	KF822534
Tetrazygia discolor (L.) DC.	Dominica, *Penneys 1287* (FLAS)‡	KF821363	EF418920	KF820544	KF822536
Tetrazygia elaeagnoides (Sw.) DC.	Ocampo and Almeda [31]	KF821365	AY460546	KF820546	KF822538
Tetrazygia fadyenii Hook.	Jamaica, *Harris 9234* (NY)*	KF821367	AY460545	KF820548	KF822540
Tetrazygia lanceolata Urb.	Cuba, *HFC 82431* (HAJB)‡	KF821368	EU055894	KF820549	KF822541
Tetrazygia longicollis Urb. & Cogn.	Dominican Republic, *Judd 8147* (NY)‡	KF821370	KF821769	KF820551	KF822543
Tetrazygia urbaniana (Cogn.) Croizat ex Mosc.	Ocampo and Almeda [31]	KF821373	EF418921	KF820554	KF822546
Tococa aristata Benth.	Guyana, *Pipoly & Boyan 8533* (CAS)*	KF821375	KF821773	KF820556	KF822548
Tococa caquetana Sprague	Ocampo and Almeda [31]	KF821379	AY460550	KF820561	KF822553
Tococa coronata Benth.	Venezuela, *Castillo 1570* (CAS)*	KF821383	AY460552	KF820565	KF822557
Tococa guianensis Aubl.	Brazil, *Rabelo et al. 3719* (CAS)*	KF821385	AY460554	KF820567	KF822559
Tococa macrophysca Spruce ex Triana	Ocampo and Almeda [31]	KF821386	AY460555	KF820568	KF822560
Tococa macrosperma Mart.	Venezuela, *Michelangeli 283* (NY)‡	KF821387	AY460556	KF820569	KF822561
Tococa nitens (Benth.) Triana	Ocampo and Almeda [31]	KF821388	AY460557	KF820570	KF822562
Tococa perclara Wurdack	Ocampo and Almeda [31]	KF821389	AY60558	KF820571	KF822563
Tococa platyphylla Benth.	Ocampo and Almeda [31]	KF821390	EU055896	KF820572	KF822564
Tococa quadrialata (Naudin) J.F.Macbride	Ocampo and Almeda [31]	KF821391	EF418922	KF820573	KF822565
Tococa rotundifolia (Triana) Wurdack	Ocampo and Almeda [31]	NA	AY460560	KF820575	KF822567
Tococa spadiciflora Triana	Ocampo and Almeda [31]	KF821394	EU055897	NA	KF822568
Tococa subciliata (DC.) Triana	Ocampo and Almeda [31]	KF821396	AY460561	KF820579	KF822571
OUTGROUP					
Adelobotrys adscendens (Sw.) Triana	Panama, *Penneys & Blanco 1665* (CAS)*	NA	AY460446	KF819860	KF821780
Adelobotrys permixta Wurdack	Guyana, *Clarke et al. 5376* (CAS)*	NA	KF821399	KF819862	KF821782
Axinaea grandifolia (Naudin) Triana	Venezuela, *King et al. 10459* (CAS)*	KF820585	AY460448	KF819867	KF821787
Eriocnema fulva Naudin	Baumgratz [21]	KF820735	EF418811	KF819990	KF821935
Graffenrieda bella Almeda	Panama, *Mori et al. 7581* (CAS)*	NA	EF418812	NA	NA
Graffenrieda gracilis (Triana) L.O.Williams	Bolivia, *Solomon 14812* (CAS)*	KF820740	KF821478	KF819995	KF821940

Graffenrieda intermedia Triana	Venezuela, *Liesner 17808* (CAS)*	KF820743	EU055684	KF819998	KF821943
Graffenrieda latifolia (Naudin) Triana	Venezuela, *Steyermark 120932* (NY)‡	KF820744	AY460450	NA	NA
Graffenrieda limbata Triana	Peru, *Foster et al. 10579* (CAS)*	KF820745	KF821481	KF819999	KF821944
Graffenrieda miconioides Naudin	Peru, *Schunke 12554* (CAS)*	KF820746	KF821482	KF820000	KF821945
Graffenrieda micrantha (Gleason) L.O.Williams	Panama, *McPherson 11228* (CAS)*	KF820747	KF821483	KF820001	KF821946
Graffenrieda moritziana Triana	Venezuela, *Meier 659* (CAS)*	KF820749	AY460451	NA	NA
Graffenrieda sessilifolia Triana	Venezuela, *Huber 10301* (CAS)*	KF820750	AY460452	KF820003	KF821948
Macrocentrum cristatum (DC.) Triana	Guyana, *Prevost & Grenand 1056* (CAS)*	NA	KF821522	KF820086	KF822038
Macrocentrum droseroides Triana	Guyana, *Clarke et al. 5451* (CAS)*	KF820852	KF821523	KF820087	KF822039
Macrocentrum fasciculatum (DC.) Triana	Guyana, *Maas & Westra 4440* (CAS)*	NA	KF821524	KF820088	KF822040
Macrocentrum minus Gleason	Guyana, *Henkel & Williams 2158* (CAS)*	KF820854	KF821526	KF820090	NA
Macrocentrum repens (Gleason) Wurdack	Guyana, *Mutchnick et al. 114* (CAS)*	KF820856	KF821528	KF820092	KF822043
Meriania involucrata (Desr.) Naudin	NA, *García 971* (NY)‡	KF820880	EF418874	KF820116	KF822066
Meriania longifolia (Naudin) Cogn.	Venezuela, *King et al. 10567* (CAS)*	KF820882	AY460454	NA	KF822068
Meriania macrophylla (Benth.) Triana	Panama, *Penneys & Olmos 1741* (CAS)*	KF820883	AY460455	KF820118	KF822069
Meriania phlomoide s (Triana) Almeda	NA, *Rodríguez 2075* (NY)‡	KF820884	EF418875	NA	KF822070
Meriania sclerophylla (Naudin) Triana	Guyana, *Pipoly 10870* (NY)‡	KF820887	AY460456	KF820121	KF822073
Meriania subumbellata Cogn.	Venezuela, *Michelangeli et al. 819* (CAS)*	KF820889	AY460457	KF820122	KF822075
Meriania urceolata Triana	Venezuela, *Huber 9774* (CAS)*	KF820891	AY460458	KF820124	KF822077
Physeterostemon fiaschii R.Goldenb. & Amorim	Brazil, *Amorim 4816* (NY)‡	KF821337	KF821758	KF820528	NA
