# Supplementary material for: Seed Diversity in the Tribe Miconieae (Melastomataceae): Taxonomic, Systematic, and Evolutionary Implications
Source: PLoS One. 2014 Jun 23;9(6):e100561. doi: 10.1371/journal.pone.0100561 (PMC4067357; doi:10.1371/journal.pone.0100561)
Supplement: Table S2 — Morphological data matrix. Numbers correspond to characters and character states shown in the Materials and Methods section. Notes: * = character with polymorphic data; ? = missing data; ‡ = species with a combination of character states that were estimated for the ancestral Miconieae seed; § = outgroup taxa. (PDF) [file pone.0100561.s004.pdf]

Table S2. Morphological data matrix. Numbers correspond to characters and character states shown in the Materials and Methods section. *Notes:* * = character with polymorphic data; ? = missing data; ‡ = species with a combination of character states that were estimated for the ancestral Miconieae seed; § = outgroup taxa.

Species / character	A*	B	C*	D*	E	F	G	H	I	J*	K*	L*	M	N	O	P	Q
Adelobotrys permixta §	3	0	0	7	2	0	0	1	0	1	1	0	0	0	0	0	4
Anaectocalyx bracteosa	0	0	0	0	2	0	0	1	0	1	1	0	0	0	0	0	1
Axinaea grandifolia §	1	0	0	7	1	0	0	1	0	1	1	0	0	0	0	0	2
Calycogonium bissei	0	0	0	0	2	0	1	1	?	1	1	0	0	0	0	0	3
Calycogonium glabratum	0	0	0	0	2	0	0	1	0	1	1	0	0	0	1	0	1
Calycogonium grisebachii	0	0	1	5	2	0	1	1	0	1	1	0	0	0	0	0	3
Calycogonium heterophyllum	1	0	0	?	2	1	0	1	0	0&1	1	1	0	0	0	0	1
Calycogonium lanceolatum	0&1	0	0	1&5	2	0	0	?	0	1	1	0	0	0	0	0	1
Calycogonium microphyllum	0&1	0	0	0	2	0	0	0	0	1	1	0	0	0	0	0	1
Calycogonium revolutum	0	0	0	0	1	0	1	1	1	0	1	0	1	0	0	0	3
Calycogonium rosmarinifolium	0	0	0	0	2	0	0	1	0	1	1	0	0	0	0	0	4
Charianthus alpinus	0&1	0	0	0&7	2	0	0	1	0	1	1	0	0	0	0	0	1
Charianthus corymbosus	0&1	0	0	5	2	0	1	1	0	1	1	0	0	0	0	0	1
Charianthus dominicensis	0&1	0	0	5	2	0	0	1	0	1	1	0	0	0	0	0	1
Charianthus nodosus	0	0	0	1&5	2	0	0	1	0	1	1	0	0	0	0	0	1
Charianthus purpureus	0&1	0	0	5	2	0	0	1	0	1	1	0	0	0	0	0	1
Clidemia allardii	0	0	0	0	1	0	0	0	0	0	1	0	1	1	1	1	1
Clidemia alternifolia	0	0	0	?	0	0	1	1	?	?	0	?	0	0	1	0	1
Clidemia angustilamina	0	0	0	?	2	0	0	1	0	1	1	0	0	0	0	0	1
Clidemia aphanantha	0	0	0	0	2	0	0	1	0	0&1	1	0	1	1	0	0	1
Clidemia capilliflora	0&1	0	0&1	?	2	0	1	1	0	1	1	0	0	0	0	0	1
Clidemia capitata	0	0	0	0	2	0	0	0	1	1	1	0	0	0	0	0	2
Clidemia capitellata	0	0	1	?	?	0	0	0	1	0	1	0	1	0	1	0	1
Clidemia caudata	1	0	0	1	1	0	1	1	0	1	1	0	0	0	1	0	1
Clidemia charadrophila	1	0	0	1	1	0	1	1	0	1	1	0	0	0	1	0	1
Clidemia ciliata	0	0	0	0	2	0	0	0	0	1	1	0	0	1	0	0	1
Clidemia clandestina	0	0	0	0	1	0	0	1	0	1	1	0	0	1	0	0	1
Clidemia conglomerata	1	0	0	0	1	0	0	?	?	1	1	0	0	0	0	?	1
Clidemia costaricensis	0	0	0	3&6	1	0	0	1	0	1	1	0	0	0	1	0	0
Clidemia crenulata	0	0	0	0	1	0	0	0	0	0	1	0	1	0	1	1	1
Clidemia densiflora	1	0	0&1	5	1	0	0	1	0	1	1	0	0	0	1	0	1
Clidemia dentata	0	0	0&1	?	?	0	0	1	0	1	1	0	0	0	?	0	0
Clidemia dimorphica	0	0	0	?	0	0	0	1	0	1	1	0	0	0	1	0	0
Clidemia discolor	0	0	0	0	1	0	0	1	0	1	1	0	0	0	1	0	0
Clidemia domingensis	0	0	1	0	2	0	0	0	0	1	1	0	0	1	0	0	1
Clidemia epiphytica ‡	0	0	0	0	1	0	0	1	0	1	1	0	0	0	0	0	0
Clidemia fendleri	0	0	0	0	2	0	0	1	1	1	1	0	0	1	0	0	1
Clidemia folsomii	0	0	0	?	1	0	0	1	0	1	1	0	0	0	1	0	0
Clidemia garciabarrigae	0	0	0	0&3	0	0	0	1	0	1	1	0	0	0	0	0	1
Clidemia globuliflora	0	0	0	0	1	0	0	0	1	0	1	0	1	0	0	0	1
Clidemia gracilis	0	0	0	1	3	1	0	1	1	1	0&1	0	0	0	1	0	1
Clidemia heteroneura	0	0	1	0	1	0	0	1	0	0&1	1	0&1	0	0	0	0	2
Clidemia heterophylla	0	0	0	?	0	0	0	1	0	1	1	0	0	0	1	0	0
Clidemia hirta	0	0	0	?	1	0	0	1	0	1	1	0	0	0	1	0	1
Clidemia inobsepta	0	0	1	0	2	0	0	1	0	1	1	0	0	0	1	0	0
Clidemia involucrata	1	0	0	?	2	0	1	1	?	?	0	?	0	0	1	1	1
Clidemia monantha	0	0	0	0	1	0	0	0	0	0	1	0	1	1	0	0	1

Clidemia ombrophila	0	0	0	0	2	0	0	1	0	1	1	0	0	0	0	0	1
Clidemia pittieri	0	0	0	0	2	0	0	0	0	1	1	0	0	0	1	0	1
Clidemia plumosa	0	0	0	?	2	0	0	1	0	1	1	0	0	0	1	0	0
Clidemia pustulata	0	0	0	0	0	0	0	0	1	1	1	0	1	0	1	0	0
Clidemia radicans	0	0	0	?	3	1	0	1	0	1	1	0	0	0	1	0	0
Clidemia reitziana	0	0	0	5	3	1	0	0	0	1	1	0	0	0	1	0	0
Clidemia rubra	0	0	0	0	1	0	0	1	0	1	1	0	0	1	0	0	1
Clidemia septuplinervia	0	0	0	?	2	0	0	1	0	1	1	0	0	0	0	0	0
Clidemia setosa	0	0	1	0	1	0	0	1	0	1	1	0	0	0	0	0	0
Clidemia tenebrosa	0	0	0	0	1	0	0	1	0	1	1	0	0	0	1	0	0
Clidemia trichosantha	0	0	1	?	?	0	1	1	0	0&1	2	0	0	0	1	1	0
Clidemia trinitensis	0	0	1	?	2	0	0	1	0	0&1	1	0	1	1	0	0	1
Clidemia umbellata	0	0	0	0	1	0	0	0	1	1	1	0	0	0	1	0	0
Clidemia umbrosa	0	0	0&1	0	1	0	1	1	0	1	1	0	1	0	1	1	0
Clidemia urceolata	0	0	0	?	1	0	0	1	1	0&1	1	0	1	0	1	0	1
Clidemia wrightii	0	0	0	?	3	1	0	1	?	1	1	0	0	0	0	1	1
Conostegia bigibbosa	0	0	0	0	1	0	0	0	1	1	1	0	0	0	0	0	1
Conostegia icosandra	0	0	0	0	2	0	0	0	0	1	1	0	0	1	0	0	0
Conostegia lasiopoda	0	0	0	0	1	0	0	1	0	1	1	0	0	0	0	1	0
Conostegia macrantha	0	0	0	0	1	0	0	0	1	1	1	0	0	0	0	0	1
Conostegia montana	0	0	0	?	1	0	0	1	0	1	1	0	0	0	0	0	0
Conostegia montealegrana	0	0	0	?	?	0	0	1	0	0&1	1	0	1	1	0	1	0
Conostegia oerstediana	0	0	0	0	1	0	0	0	1	1	1	0	0	0	0	0	1
Conostegia pittieri	0	0	0	0	1	0	0	1	1	1	1	0	0	0	0	0	1
Conostegia rhodopetala	0	0	0	?	2	0	0	1	0	1	1	0	0	0	0	0	1
Conostegia rufescens	0	0	0	?	1	0	0	1	0	1	1	0	0	0	0	0	0
Conostegia setosa	0	0	0	?	2	0	0	1	0	1	1	0	0	1	0	0	0
Conostegia speciosa	0	1	0	0	2	0	0	1	0	1	1	0	0	0	0	0	2
Conostegia subcrustulata	0	1	0	0	2	0	0	0	0	1	1	0	0	0	0	0	2
Conostegia superba	0	0	0	0	2	0	0	1	0	1	1	0	0	0	0	0	0
Conostegia tenuifolia	0	0	0	?	1	0	0	?	0	1	1	0	0	0	0	0	0
Conostegia xalapensis	0	1	0	1	2	0	0	0	0	1	1	0	0	0	0	0	1
Eriocnema fulva [§]	0	0	0	?	0	0	0	?	0	0	?	?	?	0	?	?	1
Graffenrieda bella [§]	0&1	0	0	7	2	0	1	1	0	1	1	0	0	1	0	0	1
Graffenrieda gracilis [§]	1	0	0	7	2	0	1	1	0	1	1	0	0	1	0	0	0
Graffenrieda intermedia [§]	1	0	0	7	2	0	1	1	?	1	1	0	0	1	0	1	1
Graffenrieda latifolia [§]	3	0	0	6	2	0	0	1	1	1	1	0	0	0	0	0	2
Graffenrieda limbata [§]	3	0	0	7	2	0	0	1	?	1	1	0	0	1	0	0	3
Graffenrieda miconioides [§]	1	0	0	7	2	0	1	1	1	1	1	0	0	0	0	0	1
Graffenrieda micrantha [§]	3	0	0	7	2	0	1	1	0	1	1	0	0	1	0	0	1
Graffenrieda moritziana [§]	3	0	0	7	2	0	1	1	?	1	1	0	0	0	0	1	3
Graffenrieda sessilifolia [§]	1	0	0	1	2	0	1	1	1	1	1	0	0	0	0	0	1
Leandra acutiflora	1	0	0	?	2	0	1	0	0	1	1	0	0	0	0	1	1
Leandra adenothrix	0	0	0	0&5	2	0	0	1	0	1	1	0	0	0	0	0	1
Leandra agrestis	0	0	0&1	0	0	0	0	0	1	0	1	0	1	0	1	0	0
Leandra aristigera	1	0	0	?	0	0	1	1	0	1	?	?	1	0	0	1	1
Leandra australis	0	0	0	5	2	0	?	1	0	1	1	0	0	0	0	0	1
Leandra barbinervis	0	0	?	?	2	0	0	0	0	1	1	0&1	0	0	0	0	2
Leandra carassana	0	0	0	0	2	0	1	1	0	1	1	0	0	0	0	0	2

Leandra chaetodon	1	0	0	?	?	0	1	1	0	1	1	0	1	0	0	1	1
Leandra clidemioides	1	0	0	5	1	0	1	1	0	1	1	0	0	0	0	0	1
Leandra coadunata	1	0	0	?	1	0	1	1	0	1	1	0	0	0	1	0	1
Leandra dichotoma	0	0	1	2	1	0	0	0	1	0	1	0	1	0	1	0	0
Leandra divaricata	0	0	0	2	0	0	0	0	1	0	1	0	1	0	1	0	0
Leandra edentula	1	0	0	?	1	0	1	1	0	1	1	0	0	0	0	0	1
Leandra fallacissima	0	0	0&1	0	2	0	0	0	0	1	1	0	0	0	0	0	1
Leandra fragilis	0	0	0	0&5	2	0	1	1	0	1	1	0	0	0	0	0	1
Leandra glandulifera	0	0	0	?	0	0	1	1	?	?	?	?	?	?	?	?	1
Leandra granatensis	0	0	0&1	2	1	0	0	0	1	0	1	0	1	0	1	0	0
Leandra humilis	0	0	0	5	2	0	0	1	0	1	1	0	0	0	0	0	1
Leandra inaequalifolia	?	0	?	?	?	0	?	?	0	1	1	0	1	0	0	0	0
Leandra ionopogon	0	0	0	5	2	0	1	1	0	1	1	0	0	1	0	0	1
Leandra lima	1	0	0&1	5	1	0	1	1	0	1	1	0	0	0	0	0	1
Leandra longicoma	0	0	0	2&3	1	0	0	0	1	0&1	1	0	1	0	1	0	0
Leandra macdanielii	0	0	0	0&3	0	0	0	0	1	0	1	0	1	0	1	0	0
Leandra melanodesma	0	0	0	0	2	0	0	0	0	1	1	0	0	0	0	0	0
Leandra mexicana	0	0	0	?	0	0	0	0	1	0&1	0	0	1	0	1	0	0
Leandra micropetala	0	0	0	?	?	0	1	1	0	1	1	0	0	1	0	0	1
Leandra nanayensis	0	0	0	?	1	0	1	1	?	?	?	?	?	0	?	?	1
Leandra nervosa	0	0	0	?	0	0	0	0	0	0&1	1	0	0	0	0	0	0
Leandra purpurascens	0	0	0	0&3	1	0	1	1	0	1	1	0	0	1	0	0	2
Leandra purpurea	0	0	0	0	2	0	1	1	?	?	?	?	?	0	?	?	1
Leandra quinquedentata	0	0	0	0&1	2	0	0	?	0	0&1	1	1	0	0	0	0	2
Leandra regnellii	0	0	0	5	1	0	0	0	0	1	1	0	0	0	0	0	1
Leandra reitzii	0	0	0	0	2	0	0	0	1	0&1	1	1	0	0	0	0	2
Leandra reversa	0	0	0	0&3	0	0	0	0	1	0	1	0	1	0	0	0	0
Leandra rhamnifolia	0	0	0	2	0	0	0	0	1	0	1	0	1	0	1	0	0
Leandra riograndensis	0	0	0	0&6	1	0	0	1	0	1	1	0	0	0	0	0	1
Leandra rufescens	0	0	0	0	2	0	1	1	0	1	1	0	0	1	1	1	1
Leandra salicina	0	0	0	?	1	0	0	1	0	1	1	0	0	0	0	0	1
Leandra secunda	0	0	0	2	0	0	0	0	1	1	1	0	1	0	1	0	0
Leandra secundiflora	0	0	0	?	0	0	0	0	1	1	1	0	1	0	0	0	0
Leandra subseriata	0	0	0	6	?	0	0	1	0	1	1	0	0	0	0	0	0
Leandra subulata [‡]	0	0	0	0	1	0	0	1	0	1	1	0	0	0	0	0	1
Leandra sulfurea	0	0	0	5	1	0	0	1	0	0&1	1	1	0	0	0	0	2
Leandra ulaei	1	0	0	1	2	0	0	?	0	1	1	0	0	0	0	0	1
Leandra xanthocoma	0	0	0	?	2	0	0	1	0	1	1	0	0	0	1	0	1
Leandra xanthostachya	0	0	0	?	2	0	0	1	0	1	1	0	0	1	0	0	1
Macrocentrum cristatum [§]	0	0	0	5	2	0	0	0	?	?	?	?	0	1	0	0	0
Macrocentrum droseroides [§]	0	0	0	3	0	0	0	0	1	0&1	2	1	2	0	0	1	0
Macrocentrum fasciculatum [§]	0	0	0	?	1	0	0	0	0	0	1	0	1	1	0	0	1
Macrocentrum minus [§]	0	0	1	3	1	0	0	0	1	1	1	0	0	0	1	1	0
Macrocentrum repens [§]	1	0	0	7	2	0	0	0	0	1	1	0	0	0	0	0	1
Maieta guianensis	1	0	0	6	1	0	0	1	0	1	1	0	0	0	1	1	1
Maieta poeppigii	0	0	0	3	1	0	0	1	0	1	1	0	0	0	1	1	1
Mecranium haemanthum	0	0	0	0	1	0	0	0	1	1	1	0	0	0	1	0	0
Mecranium multiflorum	0	0	1	0	1	0	0	0	0	1	1	0	0	0	1	0	0
Mecranium ovatum	0	0	0&1	0	1	0	0	1	1	1	1	0	1	0	1	0	1
Mecranium puberulum	0	0	0	0	2	0	0	0	1	1	1	0	1	0	1	0	1

Mecranium septentrionale	0	0	0	0	2	0	0	1	1	1	1	0	0	0	1	0	1
Meriania involucrata [§]	1	0	0	7	0	0	0	1	0	1	1	0	0	0	0	0	2
Meriania longifolia [§]	3	0	0	6	0	0	1	1	0	1	1	0	0	1	0	0	2
Meriania macrophylla [§]	1&3	0	0	6	0	0	0	1	0	1	1	0	0	1	0	0	2
Meriania phlomoides [§]	1	0	0	?	0	0	0	1	1	1	1	0	0	0	0	0	0
Meriania sclerophylla [§]	1	0	0	?	0	0	0	1	1	1	1	0	0	1	0	0	3
Meriania subumbellata [§]	3	0	0	?	?	0	0	1	1	1	1	0	0	0	0	0	3
Meriania urceolata [§]	3	0	0	?	0	0	0	1	?	1	1	0	0	1	0	0	6
Miconia aeruginosa	0	0	0&1	0	0	0	0	1	0	1	1	0	0	0	0	0	1
Miconia alata	0	0	0	6	1	0	1	0	0	1	1	0	0	0	0	0	1
Miconia alborufescens	0	0	1	0	2	0	0	0	0	1	1	0	2	0	0	0	2
Miconia aliquantula	0	0	0	0&1	0	0	1	1	0	1	1	0	0	0	0	1	0
Miconia amilcariana	0	0	1	0&3	2	0	0	1	0	0	2	1	2	0	0	0	1
Miconia amplinodis	0	0	0	0	1	0	0	1	0	0	0	?	0	0	1	0	1
Miconia appendiculata	0	0	1	0	2	0	0	1	0	0&1	2	1	2	0	?	0	4
Miconia arboricola	0	0	1	2&3	1	0	0	1	0	1	1	0	0	0	0	0	1
Miconia argentea	0	0	0&1	5	2	0	1	0	0	1	1	0	0	0	0	0	1
Miconia argyrophylla	0	0	0	0	2	0	0	0	0	1	1	1	0	0	0	0	2
Miconia aspergillaris	0	0	0&1	?	?	0	0	1	0	1	1	0	0	0	0	0	1
Miconia astroplocama	0	0	0	0&3	0	0	1	1	0	0	1	1	0	0	0	0	2
Miconia aymardii	0	0	0	0	2	0	0	0	1	1	1	0	0	0	0	0	1
Miconia baracoensis	0	0	0	0	2	0	0	1	0	1	1	0	2	0	0	0	4
Miconia barbeyana	0	0	0	?	2	0	0	1	0	1	1	0	0	0	0	0	0
Miconia benthamiana	0	0	0	0	1	0	0	1	0	1	1	0	0	0	0	1	0
Miconia biglandulosa	1	0	0	?	?	0	1	1	?	?	?	?	?	?	?	?	1
Miconia biperulifera	0	0	0	0	1	0	0	0	0	1	1	0	0	0	0	0	2
Miconia brachybotrya	0	0	1	0	1	0	0	1	0	0	1	0	0	0	0	0	1
Miconia bracteata	0	0	0	0	0	0	0	1	?	?	?	?	?	0	?	?	0
Miconia bracteolata	0	0	0	0	1	0	0	0	0	0	1	0	0	0	0	0	1
Miconia brasiliensis	0	0	1	3	2	0	0	0	0	1	1	0	2	0	1	0	4
Miconia brenesii	0&1	0	0	0&1	0	0	1	0	0	1	0	0	0	0	1	0	0
Miconia brevitheca	0	0	0	0	1	0	0	0	0	1	1	0	1	0	0	0	0
Miconia brunnea	0	0	1	0&2	2	0	0	0	0	1	1	0	2	0	0	0	7
Miconia bubalina	0	0	0	0&3	1	0	0	1	0	1	1	0	0	0	1	0	1
Miconia buddlejoides	0	0	1	?	?	0	0	0	0	1	1	0	0	0	0	0	4
Miconia bullata	0	0	0	0	1	0	0	0	0	1	1	0	0	0	0	0	1
Miconia cabucu	0	0	0&1	0	2	0	0	0	0	1	1	0	0	0	1	0	4
Miconia calvescens	0	0	0	3	1	1	0	1	0	1	1	0	0	0	0	0	1
Miconia calycina	0	0	0	0&1	1	0	0	0	0	1	1	0	0	0	0	0	1
Miconia campestris	0	0	0	0&3	0	0	0	0	0	0	1	0	1	0	1	0	1
Miconia caudigera	0	0	1	0	2	0	0	0	0	1	1	0	0	0	0	0	4
Miconia cerasiflora	0	0	0&1	5	1	0	0	0	0	1	1	0	0	0	0	0	6
Miconia cercophora	1	0	0	1	1	0	0	1	0	1	1	0	0	0	1	0	1
Miconia chamissois [‡]	0	0	0	0	1	0	0	1	0	1	1	0	0	0	0	0	1
Miconia chartacea	2	0	1	2	0	0	0	0	0	1	1	0	0	0	0	0	6
Miconia chrysophylla	0	0	1	0	1	0	0	0	0	1	1	0	0	0	1	0	5
Miconia ciliata	0	0	1	?	2	0	0	1	0	1	1	0	0	0	1	0	0
Miconia cinerascens	0	0	1	0	1	0	0	0	0	1	1	0	2	0	1	0	2
Miconia cinnamomifolia	0&1	0	0&1	?	2	0	0	0	0	1	1	0	0	0	0	0	2
Miconia collatata	0	0	1	0	2	0	0	0	0	1	1	0	2	0	0	0	5

Miconia concinna	0	0	0	0	1	0	0	0	0	1	1	0	0	0	0	0	1
Miconia corymbiformis	0	0	0	3	2	0	0	1	?	?	?	?	?	0	1	?	1
Miconia costaricensis	0	0	0	0	1	0	0	0	0	1	1	0	0	0	0	0	1
Miconia crocata	0	0	0	0	2	0	0	?	?	?	?	?	?	?	?	?	0
Miconia crocea	0	0	0	0	1	0	0	0	0	0	2	1	2	0	0	0	1
Miconia cubatanensis	0	0	1	?	?	0	0	0	0	0&1	1	0	2	0	1	0	4
Miconia cubensis	0	0	0	0	1	0	0	1	1	1	1	0	0	0	0	0	1
Miconia dapsiliflora	0	0	?	0	2	0	0	1	0	1	1	0	0	0	0	0	1
Miconia delicatula	0	0	0	1	2	0	0	0	0	0&1	1	0&1	2	0	1	0	3
Miconia denticulata ‡	0	0	0	0	1	0	0	1	0	1	1	0	0	0	0	0	0
Miconia desportesii	0	0	0	0	2	0	0	0	0	1	1	0	0	0	0	0	1
Miconia dielsiana	0	0	0	0&5	1	0	1	0	1	1	1	0&1	0	0	0	0	1
Miconia discolor	0	0	0&1	?	1	0	0	0	0	1	1	0	0	0	0	0	3
Miconia dispar	0&1	0	0	0&1	1	0	1	0	0	1	1	0	0	0	0	0	2
Miconia dissita	0	0	0&1	0	2	0	0	1	?	?	?	?	?	?	?	?	0
Miconia dodecandra	1	0	0	0	1	0	0	1	0	1	1	0	0	0	1	0	2
Miconia dolichopoda	0	0	0	0	1	0	0	0	0	1	1	0	0	0	0	0	2
Miconia dolichorrhyncha	0	0	0	0	2	0	0	0	0	1	1	0	2	0	1	0	2
Miconia doriana	0	0	1	0	1	0	0	0	0	0&1	1	0	0	0	1	0	4
Miconia duckei	0	0	0	0&3	1	0	1	0	0	1	1	0	0	0	0	1	0
Miconia elvirae	0	0	0	0	0	0	0	?	?	?	?	?	?	?	?	?	1
Miconia ernstii	0	0	1	0	0	0	0	1	0	1	1	0&1	0	0	0	0	3
Miconia fasciculata	0	0	1	?	2	0	0	0	0	0&1	1	0	0	0	0	0	2
Miconia ferruginea	0	0	0	0	1	0	0	0	0	1	1	0&1	0	0	0	0	1
Miconia floribunda	0	0	1	?	?	0	0	0	0	0&1	1	0&1	0	0	0	0	1
Miconia foveolata	0	0	0	0	2	0	0	1	0	1	1	0	0	0	0	0	1
Miconia friedmaniorum	0&1	0	0	0&1	1	0	1	0	0	1	2	0	2	0	0	0	1
Miconia furfuracea	1	0	0	3&5	1	0	0	1	0	1	1	0&1	0	0	0	0	2
Miconia glandulifera	0&1	0	0	0	0	0	0	1	0	1	1	0	0	0	0	0	1
Miconia goniostigma	0	0	0	0&5	2	0	0	1	0	1	1	0	0	0	0	0	1
Miconia gratissima	0	0	0	1&5	1	0	1	1	0	1	2	0	0	0	0	0	2
Miconia hemenostigma	0	0	0	0	1	0	0	0	0	1	1	0	0	0	0	0	1
Miconia holosericea	0	0	0	2&3	1	0	0	1	0	1	1	0	0	0	0	0	2
Miconia hookeriana	0	0	0&1	0&3	2	0	0	1	0	1	1	0	0	0	0	0	1
Miconia hyemalis	0	0	1	0	1	0	0	0	0	1	1	0	2	0	0	0	4
Miconia hypoleuca	1	0	0	1	2	0	0	1	0	1	1	0	2	0	0	0	1
Miconia ibaguensis	1	0	0	0&5	2	0	0	1	0	1	1	0	0	0	0	0	1
Miconia impetiolaris	1	0	1	?	2	0	0	1	0	1	1	0	0	0	0	0	2
Miconia inconspicua	0	0	1	0	2	0	0	0	0	0	1	1	1	1	0	0	2
Miconia jahnii	0	0	0	0	1	0	0	0	0	1	1	0	0	0	0	0	1
Miconia jucunda	0	0	0	0	2	0	1	0	0	1	1	0	0	0	0	0	2
Miconia krugii	0	0	0	3	1	0	0	0	0	1	1	0	0	0	0	0	1
Miconia lacera	0	0	1	3&6	1	1	0	1	0	1	1	0	0	0	1	0	0
Miconia laevigata	0	0	0	3	1	0	0	1	0	1	1	0	0	0	0	0	1
Miconia lanceolata	0	0	0	0	1	0	0	0	1	1	1	0	2	0	1	0	2
Miconia latecrenata	0	0	1	0	2	0	0	0	0	1	1	0	0	0	0	0	1
Miconia latifolia	0	0	0	0	1	0	0	1	1	0	1	0	0	0	0	0	1
Miconia leiotricha	0	0	0	0	1	1	0	0	0	1	1	0&1	2	0	0	0	1
Miconia lepidota	0	0	1	0	2	0	0	1	0	0	1	0&1	0	1	0	0	2
Miconia ligulata	0	0	0	0	1	0	1	0	0	1	1	0	0	0	1	1	0
Miconia ligustrina	0	0	0	0	1	1	0	0	0	1	1	0&1	2	0	0	0	2

Miconia ligustroides	0	0	0&1	?	2	0	0	0	0	1	1	0	0	0	1	0	2
Miconia livida	0	0	0	0	2	1	0	0	0	1	1	0	0	0	0	0	1
Miconia longicuspis	0	0	1	0	2	0	0	0	0	0	1	0	0	0	0	0	6
Miconia longifolia	0	1	0&1	?	2	0	0	0	0	1	1	0	0	0	0	0	3
Miconia longispicata	0	0	0	0	2	0	0	0	0	0	1	0	0	0	0	0	2
Miconia loreyoides	0	0	0	0	2	0	0	1	0	1	1	0	0	0	1	0	1
Miconia luteola	0	0	0	0	1	0	0	0	0	1	1	0	0	0	0	0	2
Miconia lymanii	0	0	1	0	1	0	0	0	0	1	1	0	2	0	0	0	4
Miconia magdalenae	0	0	1	0	1	0	1	1	0	1	1	0	0	0	1	0	1
Miconia manicata	0	0	0	0	2	0	0	0	0	1	1	0	0	0	1	0	1
Miconia marginata	0	0	0&1	0	1	0	0	0	0	?	0	?	0	0	1	1	0
Miconia melanotricha	0	0	1	0&6	1	0	0	1	0	1	2	0	2	0	0	0	1
Miconia melinonis	1	0	0	1	2	0	0	1	0	1	1	0	0	0	0	0	4
Miconia minutiflora	0&1	0	1	?	2	0	0	1	0	1	1	0	0	0	0	0	1
Miconia mirabilis	1	0	0	1	2	0	0	1	0	1	0	0	0	0	1	0	2
Miconia molybdea	0	0	0	3&6	1	0	0	1	0	1	1	0	0	0	0	0	1
Miconia multispicata	1	0	0	0&5	1	0	0	0	0	1	1	0	0	0	0	0	3
Miconia nitidissima	0	0	0	0	2	0	1	0	0	1	1	0	0	0	0	0	2
Miconia nystroemii	0	0	0	0	1	0	0	1	0	1	1	0&1	0	0	0	0	1
Miconia octopetala	0	0	0	0	1	0	0	0	0	1	1	0	0	0	0	0	5
Miconia oldemanii	1	0	0	0&3	1	0	0	1	0	1	1	0	0	0	0	0	1
Miconia pachyphylla	0	0	0	0	1	0	1	0	0	1	1	0	0	0	0	0	3
Miconia papillosa	0	0	0	3	1	0	0	0	0	1	1	0	0	0	0	0	1
Miconia pepericarpa	0	0	1	?	?	0	0	0	0	1	1	0	0	0	1	0	3
Miconia phanerostila	0	0	0	0	1	0	0	1	0	1	1	0	2	0	0	0	5
Miconia plumosa	0	0	0	0&5	2	0	0	0	0	0	1	1	0	0	1	0	1
Miconia poeppigii	1	0	0	1	2	0	1	1	0	1	1	0&1	0	0	0	0	2
Miconia polyandra	0	0	0	0	1	0	0	0	0	1	1	0	0	0	0	0	2
Miconia polygama ‡	0	0	0	0	1	0	0	1	0	1	1	0	0	0	0	0	1
Miconia prasina	1	0	0	1	1	0	1	1	0	1	1	0	0	0	0	0	2
Miconia procumbens	0	0	0	0	0	0	1	1	0	1	1	0	2	0	1	1	1
Miconia pseudoaplostachya	1	0	0	1	2	0	0	0	?	?	?	?	?	?	?	?	1
Miconia pubipetala	1	0	0	1	1	0	0	1	0	1	1	0	0	0	1	0	2
Miconia pulvinata	0	0	0	3&6	1	0	0	1	0	1	1	0	0	0	1	0	2
Miconia punctata	0	0	1	0	2	0	0	0	0	1	1	0	2	0	1	0	5
Miconia pusilliflora	0	0	0	0	2	0	0	0	0	1	1	0	2	0	0	0	4
Miconia pyramidalis ‡	0	0	0	0	1	0	0	1	0	1	1	0	0	0	0	0	1
Miconia racemosa	0	0	1	0&5	1	0	0	1	0	0&1	1	0	0	0	1	0	0
Miconia ramboi	0	0	0	0	2	0	0	0	0	1	1	0	2	0	1	0	2
Miconia reducens	0	0	0	0	1	0	0	1	0	1	1	0	0	0	1	0	1
Miconia rigidiuscula	0	0	0	0	2	0	0	1	0	1	1	0	0	0	0	0	2
Miconia rimalis	0	0	1	0	1	0	0	1	0	1	2	0	2	0	0	0	3
Miconia robinsoniana	0	0	0&1	0	1	0	0	1	0	1	1	0&1	0	0	1	0	1
Miconia rosea	0	0	0	6	0	1	0	1	0	1	1	0	0	0	0	0	1
Miconia rubiginosa	0	0	0	0	2	0	0	0	0	1	1	0	0	0	0	0	2
Miconia rufescens	0	0	0	0&5	2	0	0	0	0	1	1	0	0	0	0	0	1
Miconia salicifolia	0	0	0	?	?	0	0	1	0	1	1	0&1	0	0	0	0	1
Miconia samanensis	0	0	0	0	1	0	0	1	0	0	1	1	0	0	0	0	1
Miconia sanctiphilippi	1	0	0	3&5	2	1	0	1	0	1	1	0	0	0	0	0	1
Miconia schlechtendalii	0	0	0	0	2	0	0	1	0	1	1	0	0	0	0	0	1
Miconia schlimii	0	0	0	5	2	0	0	1	0	1	1	0	0	0	0	0	1

Miconia schnellii	0	0	0	0	2	0	0	0	0	1	1	0	0	0	0	0	1
Miconia sclerophylla	0	0	1	2	2	0	0	0	0	1	1	0	2	0	0	0	3
Miconia selleana	0	0	0	5	2	0	0	0	0	1	1	1	0	0	0	0	1
Miconia sellowiana	0	0	0	0	2	0	0	1	0	1	2	0	2	0	0	0	3
Miconia septentrionalis	0	0	0	0&5	1	0	0	1	0	1	1	0	0	0	0	0	1
Miconia serrulata [‡]	0	0	0	0	1	0	0	1	0	1	1	0	0	0	0	0	1
Miconia sessilifolia	0	0	0&1	0&3	2	0	0	0	1	1	1	0	1	0	1	0	1
Miconia simplex	0	0	0	0	2	0	0	1	0	1	1	0	0	0	0	0	2
Miconia sintenisii	0	0	0	0	1	0	0	0	1	1	1	0	0	0	0	0	1
Miconia skeaniana [‡]	0	0	0	0	1	0	0	1	0	1	1	0	0	0	0	0	1
Miconia smaragdina	1	0	0	1	1	0	0	1	0	1	1	0	0	0	1	0	1
Miconia sphagnicola	0	0	0	0	1	0	0	1	1	0	1	1	0	0	0	0	1
Miconia spinulosa	0	0	1	?	?	0	0	0	0	1	1	0	0	0	1	0	0
Miconia splendens	0	0	0	0&1	2	0	0	0	0	1	1	0	2	0	0	0	2
Miconia stenobotrys	0	0	0&1	5	1	0	0	1	0	1	1	0&1	0	0	0	1	2
Miconia stenostachya	0&1	0	0&1	0&5	2	0	0	0	0	1	1	0	0	0	0	0	2
Miconia striata	0	0	0	0&5	2	0	0	1	0	1	1	0	0	0	0	0	1
Miconia subcompressa	0	0	0	0	2	0	0	0	0	1	1	0	0	0	0	0	1
Miconia superba	0	0	0	0	1	0	1	1	0	1	1	0	0	1	0	0	2
Miconia sylvatica [‡]	0	0	0	0	1	0	0	1	0	1	1	0	0	0	0	0	1
Miconia tetrandra	0	0	0	0	1	0	1	1	0	1	1	0	0	0	1	0	3
Miconia tetrastoma	0	0	1	0	1	0	0	1	0	1	1	0	1	0	0	0	1
Miconia thomasiana	0	0	0	0&1	2	0	1	0	0	1	1	0	0	0	1	0	3
Miconia tomentosa	1	0	0&1	5	1	0	0	1	0	1	1	0&1	0	1	0	0	2
Miconia trianae	0	0	0	0	2	0	0	0	0	1	1	0	2	0	0	0	2
Miconia triangularis [‡]	0	0	0	0	1	0	0	1	0	1	1	0	0	0	0	0	1
Miconia trimera	0	0	1	0&3	2	0	1	1	0	1	1	0	0	0	1	1	0
Miconia triplinervis	0	0	0	3&6	0	0	0	1	0	1	1	0	0	0	1	0	0
Miconia tristis	0	0	0&1	?	1	0	0	0	0	0&1	1	0	0	0	0	0	4
Miconia tschudyoides	0	0	1	0	?	0	1	0	0	?	0	?	0	1	0	1	1
Miconia tuberculata [‡]	0	0	0	0	1	0	0	1	0	1	1	0	0	0	0	0	0
Miconia valtheri	0	0	1	0&3	1	0	0	0	0	1	1	0	0	0	0	0	2
Miconia villonacensis	0	0	1	0	1	0	0	?	?	?	?	?	?	?	?	?	1
Miconia viscidula	0&1	0	0	7	1	0	1	1	0	1	1	0&1	0	0	0	0	2
Miconia willdenowii	0	0	0	0	1	0	0	1	0	0	1	1	2	0	1	0	7
Necramium gigantophyllum	0	0	0	0	2	0	0	1	1	0	1	0	1	1	0	0	1
Ossaea amygdaloides	0&1	0	0	5	2	0	1	1	0	0	1	0	0	0	0	0	2
Ossaea brenesii	0	0	0	0	2	1	0	1	0	1	1	0	0	0	1	0	0
Ossaea capillaris	0	0	0	3&6	1	0	0	0	1	1	1	0	0	0	1	0	0
Ossaea coarctiflora	0	0	0	?	0	0	0	0	1	1	1	0	1	0	1	0	0
Ossaea confertiflora	0	0	0&1	?	2	0	0	1	0	1	1	0	0	1	0	0	2
Ossaea congestiflora	0	0	1	5	2	0	0	1	0	1	1	0	0	0	0	0	2
Ossaea macrophylla	1	0	0	5	1	0	0	0	0	1	1	0	0	0	1	0	0
Ossaea micrantha	0	0	0	0	2	1	0	1	0	1	1	0	0	0	1	0	0
Ossaea petiolaris	0	0	1	2	0	0	0	0	1	1	1	0	1	0	1	0	0
Ossaea quadrisulca	0	0	0&1	2	0	0	0	0	1	0	1	0	1	0	1	0	0
Ossaea robusta	0	0	0	5	1	0	0	1	0	1	1	0	0	0	1	0	0
Ossaea sanguinea	0	0	0	?	2	0	0	1	0	1	1	0	0	1	0	1	1
Ossaea sparrei	1	0	0&1	?	2	0	0	1	0	1	1	0	0	0	1	0	1
Ossaea spicata	1	0	0	5	2	0	0	0	0	1	1	0	0	0	1	0	0

Ossaea turquinensis	0	0	0&1	5	2	0	1	0	0	1	1	0	0	0	1	0	1
Pachyanthus angustifolius	0	0	0&1	3	1	0	1	0	0	1	1	0	0	0	?	0	1
Pachyanthus clementis	0	0	0	5	2	0	0	0	0	1	1	0	0	0	0	0	1
Pachyanthus cubensis	0	0	1	0&5	1	0	?	0	0	0&1	?	1	2	0	0	0	3
Pachyanthus mantuensis	0	0	0	5	2	0	0	0	0	1	1	0	0	0	0	0	1
Pachyanthus mayarensis	0	0	1	?	2	0	1	1	1	0	1	0	1	0	0	0	2
Pachyanthus moaensis	0	0	1	0	2	0	0	1	0	1	1	0	0	0	0	0	3
Pachyanthus pedicellatus	0	0	0&1	5	2	0	0	0	0	1	1	0	0	0	1	0	1
Pachyanthus poiretii	0	0	0	0	2	0	0	0	1	1	1	0	0	0	0	0	1
Pachyanthus reticulatus	0	0	0	5	2	0	0	0	1	?	0	?	1	0	0	0	3
Pachyanthus tetramerus	0	0	0	?	?	0	0	1	1	1	1	0	0	0	0	0	1
Pachyanthus wrightii	0	0	0	0	2	0	0	0	0	1	1	0	0	0	0	0	1
Physeterostemon fiaschii [§]	1	0	1	?	2	0	1	1	0	1	1	0&1	0	1	0	0	0
Pleiochiton ebracteatum	0	0	1	0	1	0	0	0	1	0&1	1	0	1	0	0	1	2
Pleiochiton roseum	0	0	1	?	?	0	0	0	1	0&1	1	0	1	0	0	0	1
Pleiochiton setulosum	0	0	1	?	1	0	0	0	1	0	1	0&1	1	0	0	0	1
Sagraea fuertesii	0	0	0	0	1	0	0	1	1	0	1	0	0	0	0	0	1
Sagraea scalpta	0	0	0	3&5	1	0	1	1	1	0	1	0	1	0	0	1	1
Tetrazygia barbata	0	0	0&1	?	2	0	1	0	0	1	1	0&1	0	0	0	0	3
Tetrazygia bicolor	0	0	0	7	2	0	0	1	1	1	1	0	0	0	0	0	4
Tetrazygia coriacea	0&1	0	0&1	?	2	0	1	0	1	1	1	0	0	0	0	0	2
Tetrazygia crotonifolia	0	0	0	?	1	0	0	1	0	1	1	0	0	0	1	0	1
Tetrazygia discolor	0&1	0	0	0&5	2	0	1	1	?	1	1	0	0	0	0	1	1
Tetrazygia eleagnoides	1	0	0	5	1	0	0	0	0	1	1	0	0	0	0	0	2
Tetrazygia fadyenii	0&1	0	0	?	2	0	1	1	?	?	?	?	?	?	?	?	2
Tetrazygia lanceolata	0	0	0	1	2	0	1	0	1	1	1	0	0	0	0	0	3
Tetrazygia longicollis	0	0	0	?	?	0	0	1	0	1	1	0	0	0	0	0	2
Tetrazygia urbaniana	0	0	0	0&5	1	0	0	1	0	1	1	0	0	0	1	0	1
Tococa aristata	0	0	0	0	2	0	1	1	0	0&1	1	0	0	0	0	0	2
Tococa caquetana	1	0	0	5	1	0	1	1	0	1	1	0	0	0	1	0	0
Tococa coronata	0	0	0	0	2	0	0	1	?	?	?	?	?	?	?	?	6
Tococa guianensis	0	0	0	0	2	0	0	0	1	0	1	0	1	0	0	0	2
Tococa macrophysca	0	0	0	0&3	2	1	0	1	1	1	1	1	0	0	0	0	2
Tococa macrosperma	0	0	1	0	2	0	0	0	1	0	2	1	2	0	0	0	4
Tococa nitens	0	0	0&1	0&3	2	0	0	1	0	0	1	0&1	0	0	0	0	2
Tococa perclara	0	0	1	0&3	1	0	0	1	0	1	1	0	0	0	1	0	1
Tococa platyphylla	0	0	0&1	0	2	0	0	1	0	1	1	0	0	0	1	0	1
Tococa quadrialata	0	0	0	0	1	0	0	1	1	0	1	0&1	1	0	0	0	1
Tococa rotundifolia	0	0	0	0&3	2	0	0	1	0	0	1	0	2	0	1	0	4
Tococa spadiciflora	0	0	0	6	1	0	0	0	1	1	1	0	0	0	1	0	0
Tococa subciliata	0	0	0	0&5	2	0	0	0	1	1	1	1	1	0	1	0	5
